# Ranking Plant Network Nodes Based on Their Centrality Measures

**DOI:** 10.3390/e25040676

**Published:** 2023-04-18

**Authors:** Nilesh Kumar, M. Shahid Mukhtar

**Affiliations:** Department of Biology, University of Alabama at Birmingham, Birmingham, AL 35294, USA

**Keywords:** network, graph, ranking, centrality, plant

## Abstract

Biological networks are often large and complex, making it difficult to accurately identify the most important nodes. Node prioritization algorithms are used to identify the most influential nodes in a biological network by considering their relationships with other nodes. These algorithms can help us understand the functioning of the network and the role of individual nodes. We developed CentralityCosDist, an algorithm that ranks nodes based on a combination of centrality measures and seed nodes. We applied this and four other algorithms to protein–protein interactions and co-expression patterns in *Arabidopsis thaliana* using pathogen effector targets as seed nodes. The accuracy of the algorithms was evaluated through functional enrichment analysis of the top 10 nodes identified by each algorithm. Most enriched terms were similar across algorithms, except for DIAMOnD. CentralityCosDist identified more plant–pathogen interactions and related functions and pathways compared to the other algorithms.

## 1. Introduction

Gene and protein networks are commonly interpreted as encoding functional information in their connections. The connections within these networks can provide insights into the functions of genes and proteins and can help to understand how they contribute to cellular processes and overall biological function [1,2,3]. Network-based gene prioritization is a method of identifying genes that are likely to be important or relevant to a particular biological process or disease [4]. It involves constructing a network of interacting genes or proteins and using various algorithms to prioritize or rank the genes within the network. The concept of ‘guilt by association’ is frequently used in network-based approaches for identifying important genes. The principle behind this approach is that genes that are closely connected in a protein–gene interaction network are likely to be involved in the same functional processes and may exhibit similar characteristics or traits [5]. Various algorithms can be used for gene prioritization including centrality measures (such as degree centrality and betweenness centrality) and diffusion-based methods (such as random walk with restart (RWR)). These algorithms can be applied to various types of networks including protein–protein interaction networks (PPI), co-expression networks, and functional networks [6]. Gene prioritization can be useful for identifying potential therapeutic targets for diseases, for understanding the underlying mechanisms of complex biological processes, and for identifying potential biomarkers for diagnosis or prognosis. 

The network-based gene prioritization methods can generally be classified into four categories: guilt by association, centrality measures, network propagation/random walk, and network clustering/communality analysis. These methods-involve analyzing the connections within a network to identify genes or proteins that are considered functionally important. Each method has its own unique approach to analyzing the network and identifying key genes, and therefore each has its strengths and limitations.

- Guilt by association: This method involves identifying genes that are closely connected to known disease-associated genes in a protein–protein interaction network. The idea is that genes, which are connected to disease-associated genes are more likely to be involved in the disease process [7,8].
- Centrality measures: This method involves identifying genes that are central or highly connected in a network, as these genes may be thought to be functionally important. Examples of centrality measures include degree centrality, betweenness centrality, and closeness centrality [8,9,10,11,12,13].
- Network propagation/random walk: This method involves simulating the movement of a random walker through a network, with the idea that the walker is more likely to visit functionally important nodes in the network [14,15,16].
- Network clustering/communality analysis: This method involves identifying clusters or modules of genes within a network that are more highly connected to one another than to genes in other clusters. These clusters may represent functionally related groups of genes [17].

Moreover, there are several different types of centrality measures in network analysis [13]. These measures are used to identify the most important nodes in a network, based on the relationships and connections between the nodes. Here are some examples of different types of centrality measures:

1. Degree centrality: This measure represents the number of connections a node has to other nodes in the network. Nodes with a high degree of centrality have many connections and are considered to be central to the network [18].
2. Betweenness centrality: This centrality describes the number of shortest paths between other nodes that pass through a particular node. Nodes with high betweenness centrality are considered to be important gatekeepers in the network as they control the flow of information and resources between other nodes [18].
3. Closeness centrality: This measure is based on the average distance from a particular node to all other nodes in the network. Nodes with high closeness centrality are considered to be well-connected and able to reach other nodes quickly [19].
4. PageRank: This is a measure of the importance of a node in a directed network, based on the number and quality of incoming links to the node. It was developed by Google as part of its search algorithm and is used to rank web pages [18].
  a. Personalized PageRank is a variant of the PageRank algorithm that takes into account the specific interests or preferences of an individual user. In the standard PageRank algorithm, the importance of a node is based on the number and quality of incoming links to the node. However, in personalized PageRank, the importance of a node is also based on the interests of the user.
5. Information centrality: This centrality measure determines the importance of a node in a network based on the flow of information through the node. It is used to identify the most important nodes in a network in terms of the transmission of information [20].
6. Eigenvector centrality: This centrality defines the importance of a node in a network based on the concept that a node is important if it is connected to other important nodes. It is used to identify the most influential nodes in a network based on the connections between nodes. The eigenvector centrality of a node is calculated by taking into account the eigenvectors (a set of vectors that are scaled versions of the original vectors) of the connections between the nodes in the network. The eigenvectors are used to determine the relative importance of the nodes, with more important nodes having higher eigenvector centrality scores [19,20].
7. Clustering coefficient: The clustering coefficient centrality of a node in a network is a measure of the node’s importance based on the number of triangles it is a part of. A triangle is a group of three nodes that are all connected to each other, forming a loop. The clustering coefficient centrality of a node is calculated by dividing the number of triangles the node is a part of by the total number of possible triangles it could be a part of. A node with a high clustering coefficient centrality score is considered to be important because it is connected to many other nodes that are also connected to each other, forming a densely connected cluster within the network [20].

Centrality measures are often used in network analysis to identify the most important nodes in a network based on the connections and relationships between the nodes. However, these measures may not always perform well in gene prioritization tasks, which involve identifying the most important genes in a network based on their relationships to other genes and their potential role in a particular biological process.

There are several reasons why centrality measures may not perform well in gene prioritization [21]. Centrality measures are based on the connections between nodes and do not take into account the specific characteristics or functions of the nodes themselves. In gene prioritization, the specific functions and characteristics of the genes are often more important than their connections to other genes. Centrality measures do not always accurately capture the complexity of the relationships between genes in a network. Gene networks are often highly complex and dynamic, with many different types of interactions and relationships between genes. Simple centrality measures may not be able to accurately reflect the importance of genes in such networks. Centrality measures are sensitive to the size and structure of the network. In gene networks, the size and structure of the network can vary widely depending on the specific biological process being studied. This can make it difficult to accurately compare the importance of genes across different networks. Overall, while centrality measures can be useful for identifying the most important nodes in a network, they may not always perform well in gene prioritization tasks due to the complexity and diversity of gene networks.

Furthermore, it is possible to use a combination of centrality measures for node prioritization, in order to incorporate multiple aspects of a gene’s importance and influence within a network. By combining multiple centrality measures, it may be possible to obtain a more comprehensive view of a gene’s role and importance in the network [22].

For example, one approach could be to use a combination of degree centrality, which measures the number of connections a gene has to other genes in the network, and eigenvector centrality, which measures the influence of a gene based on the influence of the genes it is connected to. This could provide a more complete picture of a gene’s importance, taking into account both its direct connections to other genes and its indirect influence through its connections to other influential genes.

It is also possible to use machine learning techniques to combine multiple centrality measures and other types of data such as gene expression data or functional annotation data to predict the importance of genes in a network. By training a machine learning model on a large dataset of known important genes, it may be possible to use the model to predict the importance of novel genes in a network.

Overall, using a combination of centrality measures and other types of data is poised to be a powerful approach for node prioritization, as it allows for a more comprehensive evaluation of a node’s importance and role in a network [23]. 

Our proposed method, CentralityCosDist, uses a combination of nine centrality measures to prioritize nodes in the PPI and co-expression network: degree centrality, betweenness centrality, closeness centrality, eigenvector centrality, PageRank, personalized PageRank, information centrality, eigenvector centrality, and clustering coefficient. To begin, a network centrality analysis is conducted on a given network that includes seed nodes. In network biology, a seed node is a starting point for identifying other nodes in a network that are related to the seed node through some criteria of interest. For example, a seed node might be a protein that is known to be involved in a particular biological pathway, and the goal might be to identify other proteins that are also part of that pathway. To do this, one might use the seed node as a starting point and then search the network for other nodes that are connected to the seed node. This can be done using algorithms that traverse the network and look for connections between nodes. The resulting group of interconnected nodes can then be analyzed to better understand the function and organization of the pathway or other biological processes of interest.

By using centrality analysis, the nodes in the network are expressed as 9-dimensional vectors. Cosine distances between seed nodes and all other nodes were computed using the centrality vectors. The mean distance between the seed and other nodes was used to determine the ranking of the nodes. Four different prioritization algorithms—DIAMOnD [24], GenePANDA [25], Node2Vec [26,27], and RWR [28]—were applied to all networks to identify the vital nodes in the network by using the same seed node sets for all networks [6].

## 2. Materials and Methods

Multiple co-expression and PPI networks were utilized in this study. A total of 16 co-expression networks related to development processes [29], two co-expression networks related to pathogens [30,31], eight PPI networks from STRING, and three experimental PPI networks were included in this study. All co-expression networks used in study were WGCNA-based co-expression networks [32]. In addition, two sets (HarTAIRdef17 and HopTAIRdef17) of 17 seed nodes related to plant pathogen effector targets were selected. Our method, CentralityCosDist, combines nine centrality measures to prioritize nodes in PPI and co-expression networks, including degree centrality, betweenness centrality, closeness centrality, eigenvector centrality, PageRank, Personalized PageRank, information centrality, eigenvector centrality, and clustering coefficient. The network centrality analysis was conducted using the NetworkX (2.8.8) Python package. For the calculation of personalized PageRank, seed nodes present in the network were used as personalized vectors. Centrality analysis was used to express nodes in the network as 9-dimensional vectors, and cosine distances between the seed nodes and all of the other nodes were calculated using these centrality vectors. 

The cosine distance is a measure of similarity between two non-zero vectors of an inner product space. It is defined as the cosine of the angle between the two vectors, which is also equal to the dot product of the vectors divided by the product of their magnitudes. The cosine distance is commonly used in information retrieval and machine learning to compare the similarity of documents, words, or other data points represented as vectors. To measure the similarity between centrality vectors, we used scikit-learn’s cosine_similarity function. The cosine_similarity function returns a similarity matrix with dimensions (n_samples_1, n_samples_2), where each element (i, j) represents the cosine similarity between the ith element of the first set and the jth element of the second set. The elements of the similarity matrix range from −1 to 1, with a value of 1 indicating that the vectors are identical, and a value of −1 indicating that the vectors are completely dissimilar.

**Figure 1:**
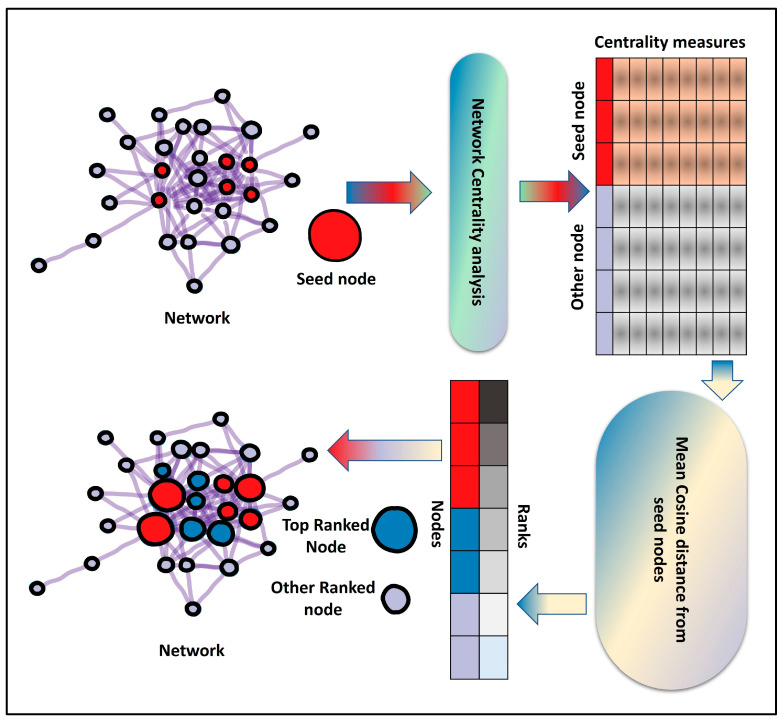
The overall workflow of CentralityCosDist.

To rank the nodes, we used the mean cosine similarity between the seed nodes and other nodes. Furthermore, four prioritization algorithms—DIAMOnD, GenePANDA, Node2Vec [26,27], and RWR [28]—were also applied to all networks to identify the key nodes by using the same seed node sets for all networks. The overall workflow of CentralityCosDist is summarized in the graphical abstract (Figure 1) and the is the pseudo code of CentralityCosDist is as follows:

**Step 1**: *Perform multiple network centrality analyses on the network*.

**Step 2**: *Determine the cosine similarity among seeds by using centrality measures as vectors.*

**Step 3**: *(Optional) eliminate seeds that are highly dissimilar to the majority of the other seeds.*

**Step 4**: *Determine the cosine similarity between the chosen seed and all other nodes.*

**Step 5**: *Calculate the mean similarity of all nodes from all seed nodes.*

**Step 6**: *Rank all nodes based on the mean cosine similarity score.*

To assess the ranking performance of each algorithm, we separately investigated the ranking of seed sets for each algorithm. We compiled all nodes that were ranked within the top 10 by each algorithm, irrespective of the network they belonged to. For qualitative assessment of the rankings produced by the different methods, we used the same set of top-ranked genes to perform functional enrichment analysis using Metascape [33].

## 3. Results

An essential application of ranking nodes within a biological network is to uncover genes or proteins that are related to a gene set of seed nodes involved in a biological process, pathway, or disease. Therefore, a successful ranking method should be able to place highly relevant genes or proteins at the top of the list of genes or proteins that are similar or relevant to the given seed nodes. CentralityCosDist uses indicators of centralities to encode nodes as vectors and calculates the cosine similarity between the nodes and seed nodes. 

Many different indicators of centrality can be used to measure the importance or influence of a node in a network. Some commonly used centrality measures include degree centrality, betweenness centrality, closeness centrality, eigenvector centrality, and PageRank centrality. Diverse centrality measures can highlight various aspects of a network and identify different types of important nodes. For example, degree centrality is useful for identifying nodes that have a large number of connections and are therefore well-connected in the network. Betweenness centrality is valuable for identifying nodes that lie on many shortest paths and can control the flow of information in the network. Closeness centrality is suitable for identifying nodes that are close to all other nodes and can reach other nodes quickly. Eigenvector centrality is advantageous for identifying nodes that are connected to many other important nodes. PageRank centrality is important for identifying nodes that have many inbound links from other important nodes. Each centrality measure has its unique perspective on the network and can be useful for different purposes. To determine which centrality measure would be most effective for prioritizing important nodes, we used an unsupervised labeling method to add labels to the nodes. We used the Density-based spatial clustering of applications with noise (DBSCAN) and K-means clustering algorithms to cluster the nodes in the network. The DBSCAN algorithm is a density-based clustering method that can identify clusters of different sizes and shapes, while the K-means algorithm is a centroid-based method that divides the data into a predefined number of clusters. We applied these algorithms to the nodes in the network to group them into different clusters and gain insights into the underlying structure of the network. After clustering the nodes, we used the random forest algorithm to calculate the feature/centrality importance scores. The random forest algorithm is an ensemble learning method that creates a large number of decision trees and combines them to make a prediction. By training a random forest model on the data, we were able to calculate the feature importance scores, which reflect the relative importance of each feature in predicting the target variable. This allowed us to identify the most important centrality in the data and understand how they contribute to the overall prediction. 

We used the feature selection strategy to learn the importance of centralities. Using DBSCAN and K-means algorithms, nodes of each network were clustered based on the network centralities. Furthermore, the permutation importance evaluation technique was used to measure the effect of randomly permuting a centrality (feature) on the random forest model performance as it provides an estimate of how important each centrality is for the predictions of the model. If centrality is important, then shuffling its values should have a noticeable effect on the model’s performance. A high score for centrality indicates that it is an important feature of the model, and a low score suggests that it is not as important. We found that for different networks, different centrality measures played important roles in predicting the node labels (DBSCAN and K-means cluster node). However, overall, we observed that the closeness centrality, eigenvector centrality, and informational centrality measures were particularly effective (Figures S1 and S2).

It is worth noting that personalized PageRank was not used for this analysis because the concept of seed nodes is not applicable at this stage.

**Figure 2:**
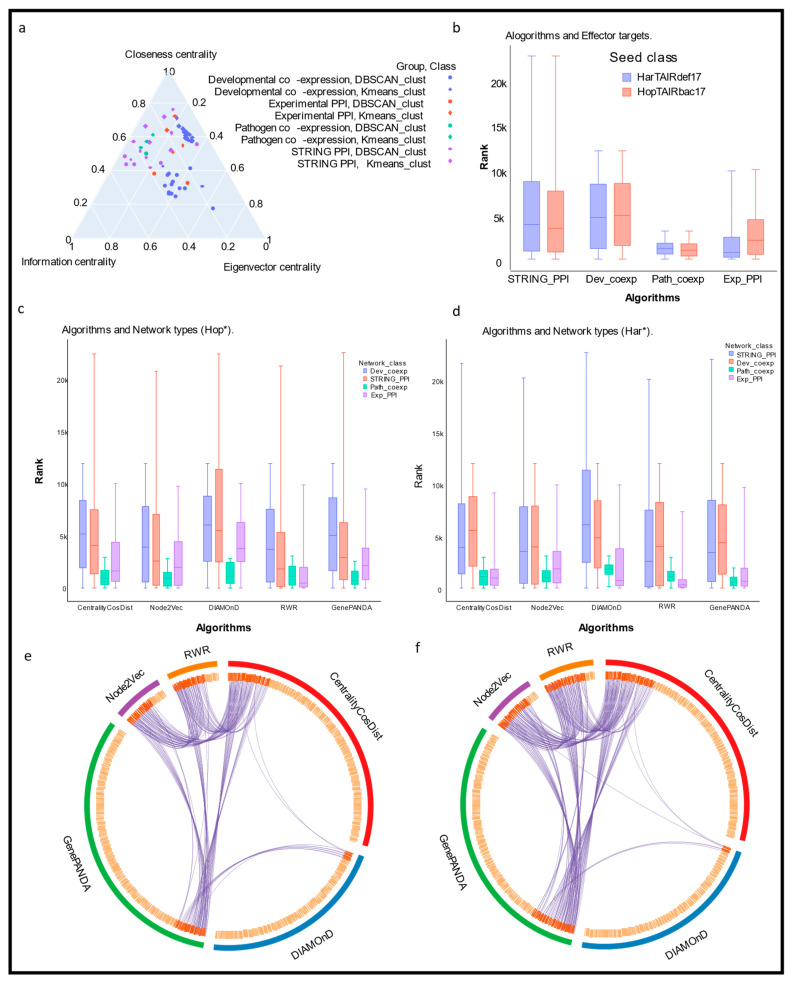
Effectiveness of CentralityCosDist. (**a**) A ternary plot to illustrate the effectiveness of centrality measures for different networks and their respective clustering algorithms used for creating unsupervised labels. In the ternary plot, the three sides represent different centrality measures: closeness centrality, eigenvector centrality, and information centrality. Data points from various network groups, along with their corresponding clustering algorithms, are plotted using different shapes (DBSCAN: circle, K-means: diamond) and colors (developmental co-expression: blue; experimental PPI: red; pathogen co-expression: green; STRING PPI: violet). (**b**) Ranking of seed class in diverse networks. The boxplot of the distribution of rankings for effector proteins (Har: blue and Hop: red) generated by various algorithms. The rankings are combined across all networks to provide a comprehensive analysis. (**c**,**d**) The boxplot illustrate the distribution of rankings for effector proteins (Har: blue and Hop: red) generated by various algorithms across different network groups, including developmental co-expression (blue), experimental PPI (violet), pathogen co-expression (green), and STRING PPI (red). (**e**,**f**) The chord diagram shows the overlapping top-ranked genes across the network as identified by different algorithms. The rankings across all networks are pooled to provide a comprehensive insight.

We used a ternary plot to illustrate the effectiveness of the closeness centrality, eigenvector centrality, and informational centrality measures for the different networks and the respective clustering algorithms used for creating unsupervised labels (Figure 2a). A ternary plot is a graphical representation of three variables that sum to a constant. In a ternary plot, the three variables are represented by the three sides of an equilateral triangle, with the lengths of the sides proportional to the values of the variables. The vertices of the triangle represent the three possible combinations of the centralities, and the positions of the data points within the triangle represent the relative values of the variables. Round dots and diamond-shaped dots represent the DBSCAN and K-means, respectively, whereas the four colors in the plot represent the different types of networks that were analyzed. 

After that, we applied all five ranking methods including CentralityCosDist, to each network twice using both seeds (HarTAIRdef17 and HopTAIRdef17) separately. We combined all of the rankings of each network-by-network group (Dev_coexp, Path_coexp, STRING_PPI, Exp_PPI). Overall, we observed that the set of 17 seeds was more densely ranked in the pathogen co-expression network (Path_coexp) compared to the other network groups (Figure 2b) and existing algorithms (Figure 2c,d).

**Figure 3:**
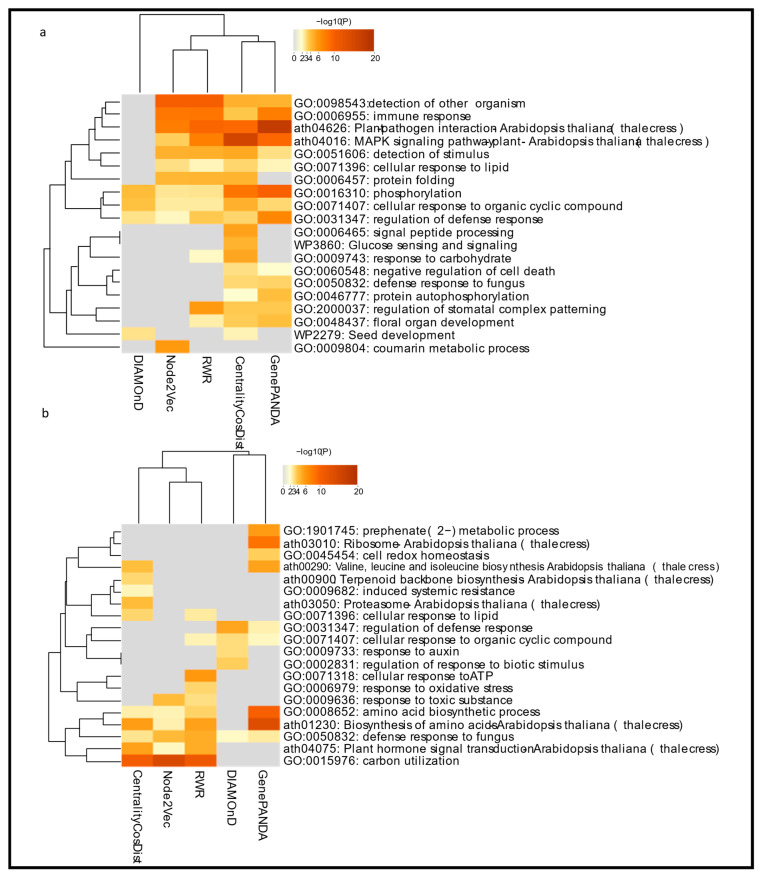
Functional enrichment analysis using Metascape in (**a**) Hop and (**b**) Har groups.

We also investigated the ranking of seed sets separately for each algorithm (Figure 2e,f). The range of ranks of both seed groups generated by CentralityCosDist agreed with the range of ranks generated by the other methods. We then compiled all nodes that were ranked 10 or lower by each algorithm, regardless of the network they belonged to. The overlap among the top-ranked nodes by all five methods is displayed with the help of a chord diagram (Figure 2e,f). For the qualitative assessment of the rankings produced by the different ranking methods, we used the same set of top-ranked genes to perform functional enrichment analysis using Metascape (Figure 3a,b). It is evident that the pathways relevant to plant–pathogen interaction and defense, which are highly significant, were consistently enriched with all methods. In contrast, the functional and pathway enrichment did not consistently align with all methods including CentralityCosDist. 

## 4. Discussion

Our proposed network centrality-based node prioritization method involves representing nodes in a vector form using various indicators of centrality and then calculating the cosine similarity between the non-seed node vectors and the vectors of the seed nodes. This allowed us to rank the nodes in a way that prioritizes those that are most relevant to the seed nodes. We tested this method on a variety of different networks and found it to be effective in identifying key nodes in co-expression and protein–protein interaction networks. 

There are many potential applications for a network centrality-based method for prioritizing nodes in a network such as identifying disease-associated genes, characterizing functional modules in a network, identifying key players in a network, and prioritizing targets for drug discovery. In our study, we compared the results of our node prioritization method CentralityCosDist with those from four widely accepted methods: DIAMOnD, GenePANDA [25], Node2Vec [26,27], and RWR [28] (Figure 2c,d). These methods are all commonly used for ranking nodes in a network and are effective in identifying disease-associated genes and functional modules. By comparing our results with those from these methods, we were able to gauge the performance of our method and assess its relative strengths and weaknesses. Overall, our method showed promising results and performed competitively with the other methods. 

Using a chord diagram, we were able to visualize the overlap among the top-ranked genes produced by different node ranking methods. We found that the top-ranked genes from our method quantitatively overlapped with the top-ranked genes from most of the other methods including GenePANDA [25], Node2Vec [26,27], and RWR [28]. However, the overlap with the top-ranked genes from the DIAMOnD [24] method was not as strong. This suggests that while our method is generally consistent with the other methods in terms of the genes it identifies as important, it may have some unique characteristics that differentiate it from the DIAMOnD [24] method. Further analysis will be needed to understand the reasons for this difference and to determine the relative strengths and limitations of each method. 

To assess the quality of the top-ranked genes produced by different node ranking methods, we performed functional enrichment analysis using the top-ranked genes from each method. We found that the functional enrichment results were mostly consistent among CentralityCosDist, GenePANDA [25], Node2Vec [26,27], and RWR [28]. This suggests that these methods are generally able to identify genes that are involved in important biological processes and pathways. However, there were some differences among the methods in terms of the specific pathways and functions that were enriched. Further analysis will be needed to understand the reasons for these differences and to determine the relative strengths and limitations of each method. Overall, the functional enrichment results support the validity of our proposed node ranking method and suggest that it has the potential to be a useful tool for identifying disease-associated genes or proteins and understanding the underlying biology of a system. Both seed node list contains effector protein targets. 

Effector proteins, secreted molecules by diverse microbes including pathogenic bacteria [34], can affect the physiology of their host cells to facilitate colonization and infection. These proteins target various cellular processes to manipulate the host’s behavior or function in ways that are beneficial to the bacteria. There are several cellular processes that effector proteins may target in order to manipulate the host’s behavior or function. These include processes such as cell growth and division, defense responses, hormone signaling, protein synthesis, and protein degradation. Our functional enrichment analysis supports the idea that these processes are indeed targeted by effector proteins, as we observed enrichment for genes and pathways related to these processes. 

Our results from the CentralityCosDist node ranking method identified several pathways that were uniquely exclusive to our method, meaning that these pathways were enriched in the top-ranked genes produced by CentralityCosDist but not by the other ranking methods. These pathways are known to be important for plant defense and include ‘signal peptide processing’, which is involved in the synthesis and processing of proteins that play a role in defense responses; ‘glucose sensing and signaling’, which regulates the allocation of glucose to different metabolic pathways and is involved in defense responses; ‘terpenoid backbone biosynthesis’, which is important for the synthesis of terpenoids, which have antimicrobial properties and can act as phytoalexins; ‘induced systemic resistance’, which refers to the activation of defense responses in response to certain types of stress such as pathogen attack; ‘proteasome’, which is involved in the degradation of unneeded or damaged proteins and plays a role in the activation of defense responses. These pathways may be particularly relevant to understanding the biology of plant–pathogen interactions and developing strategies for controlling plant diseases. CentralityCosDist is a network centrality-based node prioritization method that represents nodes as vectors using various measures of centrality and calculates the cosine similarity between non-seed nodes and seed nodes. Centrality-based node prioritization methods are widely used tools for identifying important or influential nodes in a network. However, there are a few potential limitations to consider when using these methods. One limitation is that centrality-based methods are sensitive to the underlying network structure and may produce different results depending on the specific network being analyzed. This means that the results of a centrality-based analysis may not be directly comparable across different networks or datasets. Another limitation is that centrality-based methods often make assumptions about the connectivity of the network, which may not always hold true in real-world networks. This can lead to inaccurate or misleading results if the assumptions are not valid for the particular network being analyzed. Additionally, centrality-based methods typically only consider pairwise relationships between nodes, which may not capture more complex interactions in the network. This can limit their ability to identify indirect relationships between nodes, which can be important for understanding the underlying biology of a system. Finally, the performance of centrality-based methods can be strongly influenced by the choice of seed nodes, which can make it challenging to compare results across different studies. To overcome the limitations of current network-based gene prioritization algorithms, future work should focus on developing approaches that are less sensitive to the underlying network structure and make fewer assumptions about network connectivity. These improvements could help to increase the performance and reliability of these algorithms, making them more useful for researchers working on network-based analyses of biological data. 

CentralityCosDist is a node prioritization algorithm that has significance in the field of biological networks. It helps to identify the most influential nodes in the network by combining centrality measures and seed nodes. By doing so, it provides a more comprehensive view of the network and the role of individual nodes. CentralityCosDist considers multiple measures of centrality and uses cosine distance, a measure of similarity, to combine them, allowing it to capture both local and global network properties. Furthermore, CentralityCosDist is robust to noise and outliers because it uses a variety of centrality measures to rank nodes. This means that it is not heavily reliant on any one measure, which makes it less susceptible to noise and outliers. In addition, CentralityCosDist can be useful for networks where the relationships between nodes are complex and multi-faceted. For example, in a biological network where different types of interactions between proteins can occur, CentralityCosDist can help identify the most influential proteins based on their various types of interactions. The algorithm has been applied to protein–protein interactions and co-expression patterns in *Arabidopsis thaliana* and has shown improved results in identifying plant–pathogen interaction functions and pathways compared to other algorithms. The results demonstrate the usefulness of CentralityCosDist in understanding the functioning of biological networks and highlighting key nodes that play important roles in the network.

## Data Availability

The script used in this work has been deposited in GitHub (https://github.com/nilesh-iiita/CentralityCosDist).

## Supplementary Materials

The following supporting information can be downloaded at: https://www.mdpi.com/article/10.3390/e25040676/s1, Figure S1: DBSCAN clustering and feature selection; Figure S2: K-Means clustering and feature selection.
