# Supplementary material for: Ranking Plant Network Nodes Based on Their Centrality Measures
Source: Entropy (Basel). 2023 Apr 18;25(4):676. doi: 10.3390/e25040676 (PMC10137616; doi:10.3390/e25040676)
Supplement: Supplementary file 1 [file entropy-25-00676-s001.zip › Figure_S2_KMEANS_feature_selection_plot.pdf]

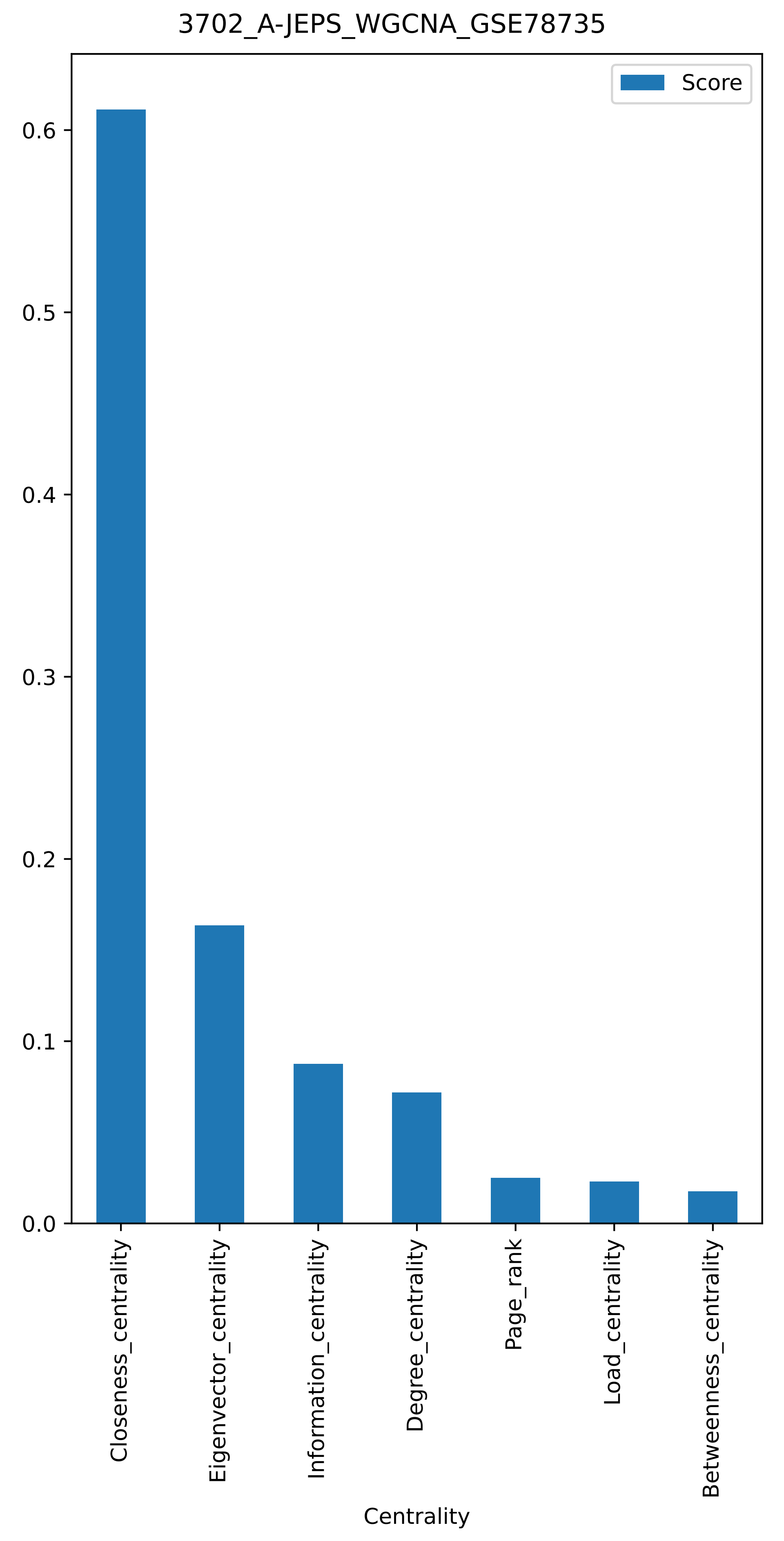
3702_A-JEPS_WGCNA_GSE78735
Score
0.0
0.1
0.2
0.3
0.4
0.5
0.6
Closeness_centrality
Eigenvector_centrality
Information_centrality
Degree_centrality
Page_rank
Load_centrality
Betweenness_centrality
Centrality

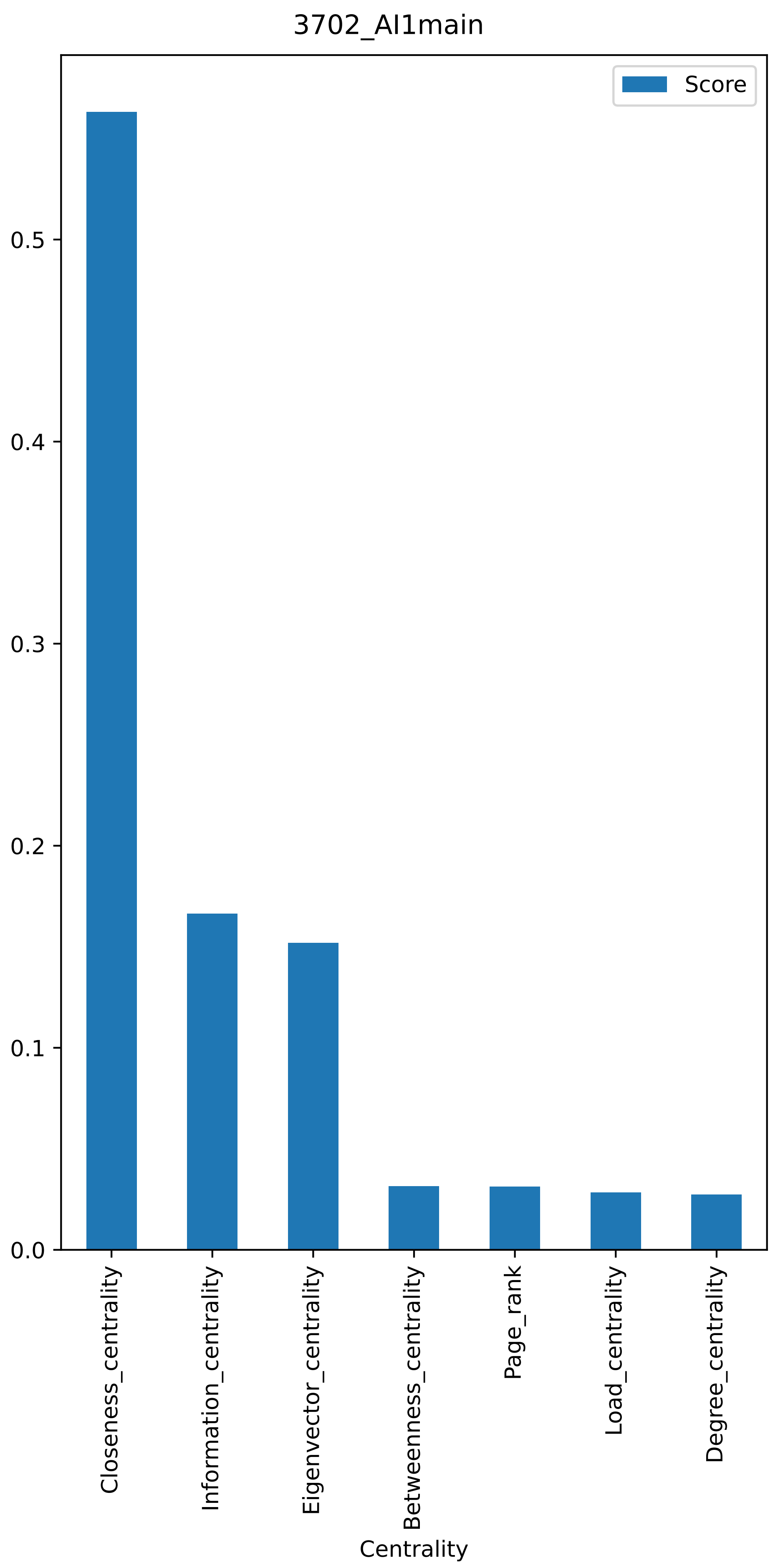

3702_AI1main
Score
0.0
0.1
0.2
0.3
0.4
0.5
Closeness_centrality
Information_centrality
Eigenvector_centrality
Betweenness_centrality
Page_rank
Load_centrality
Degree_centrality
Centrality

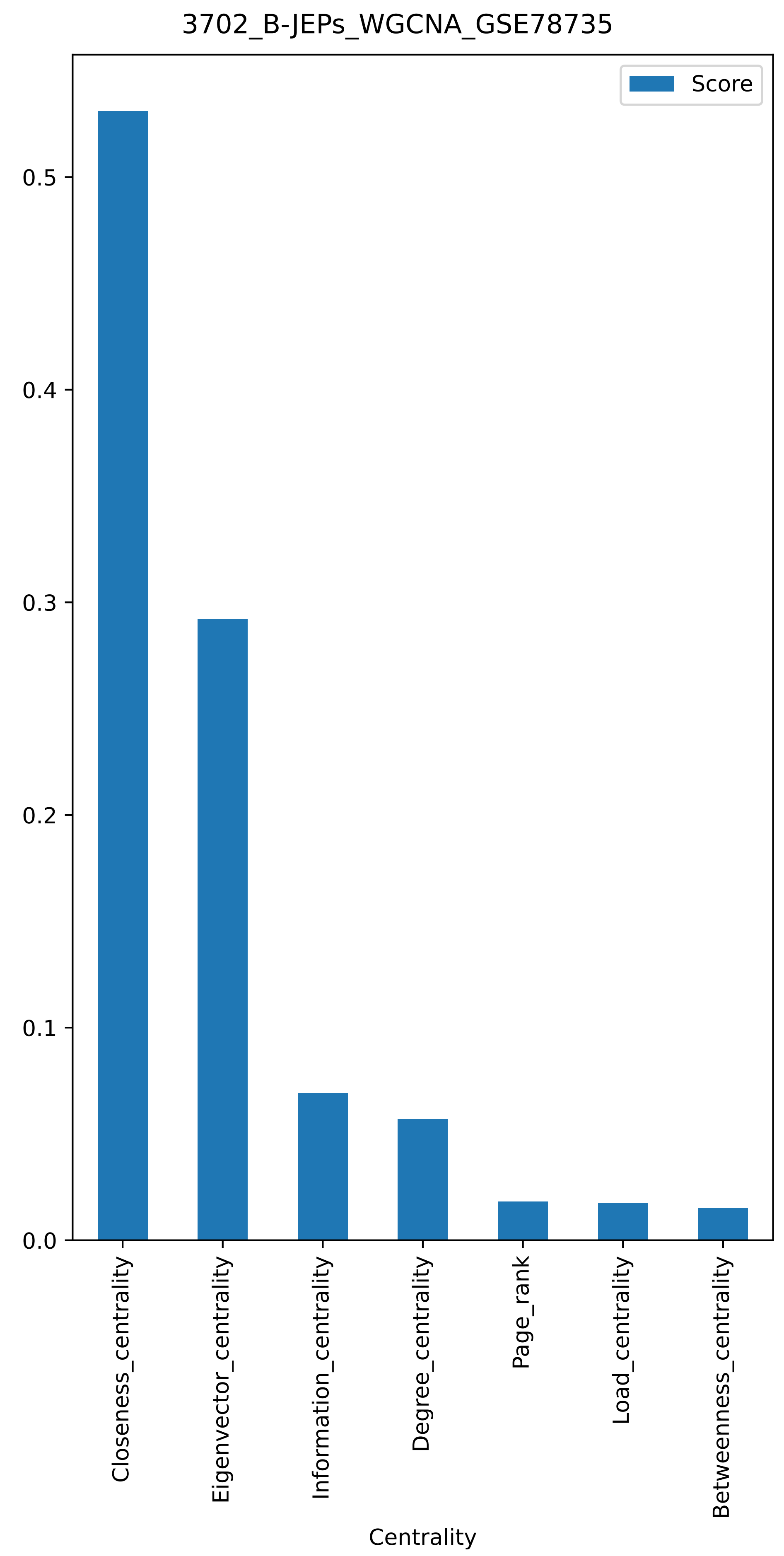

3702_B-JEPs_WGCNA_GSE78735
Score
0.0
0.1
0.2
0.3
0.4
0.5
Closeness_centrality
Eigenvector_centrality
Information_centrality
Degree_centrality
Page_rank
Load_centrality
Betweenness_centrality
Centrality

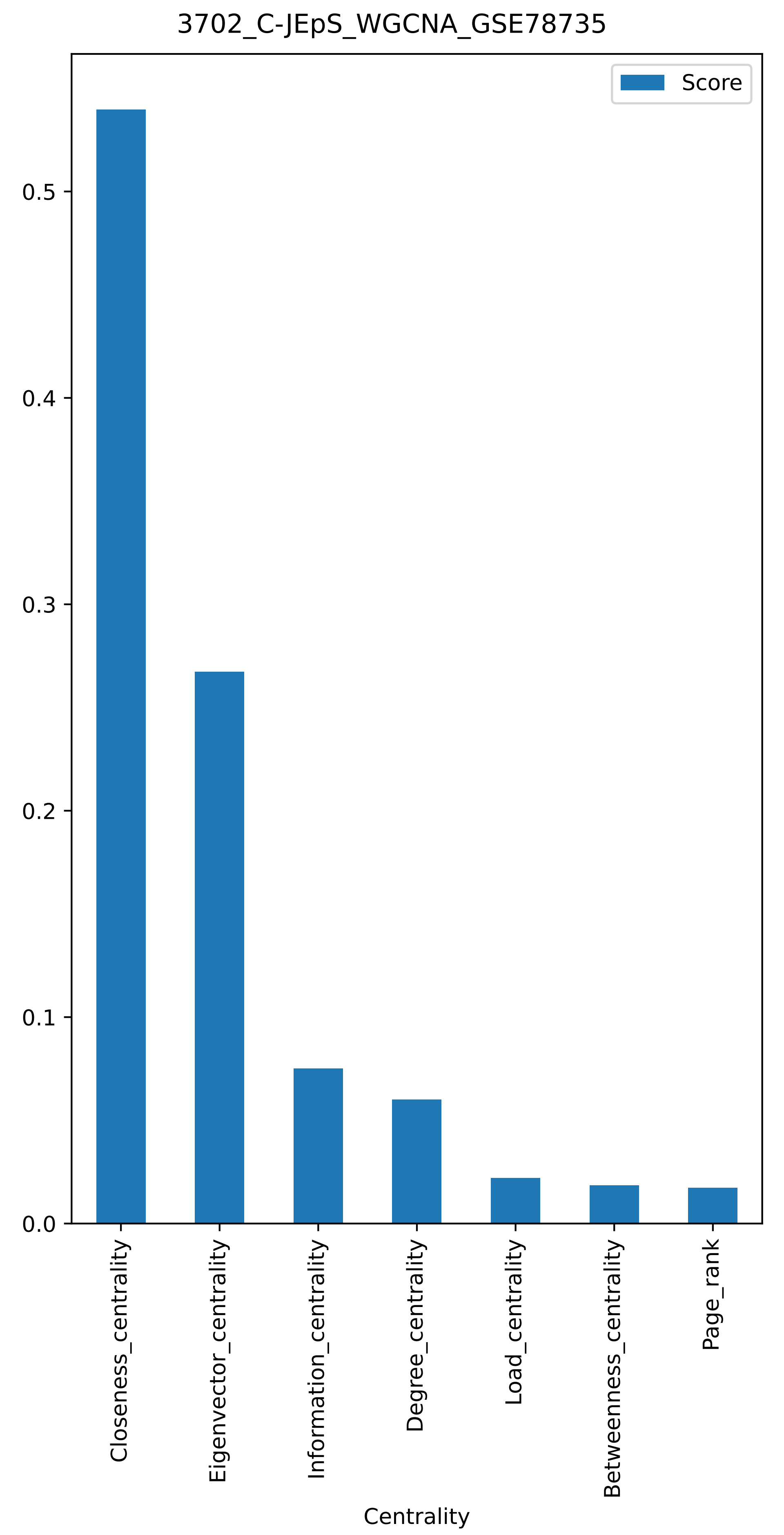
3702_C-JEpS_WGCNA_GSE78735
Score
0.0
0.1
0.2
0.3
0.4
0.5
Closeness_centrality
Eigenvector_centrality
Information_centrality
Degree_centrality
Load_centrality
Betweenness_centrality
Page_rank
Centrality

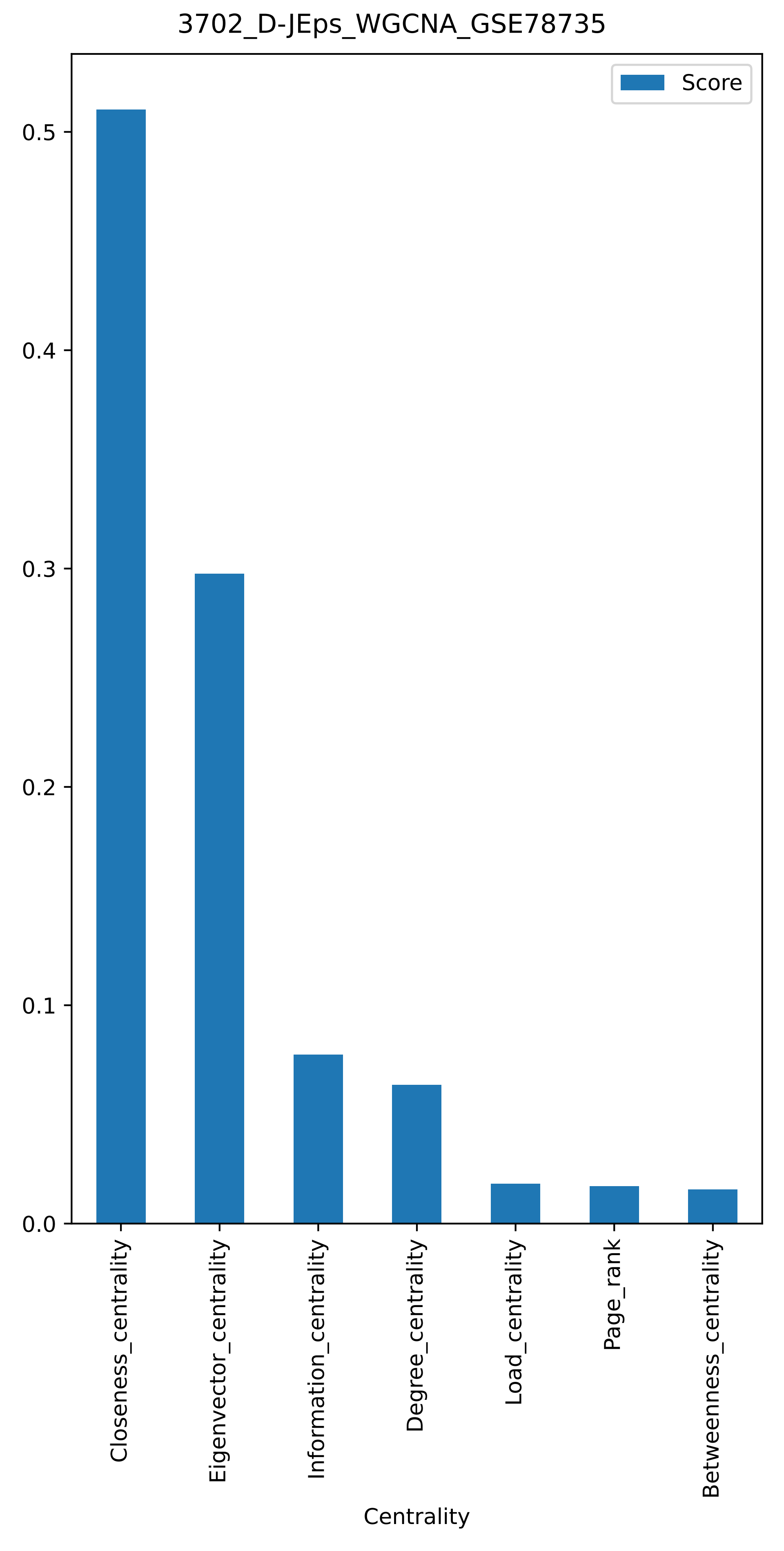
3702_D-JEps_WGCNA_GSE78735
Score
0.0
0.1
0.2
0.3
0.4
0.5
Closeness_centrality
Eigenvector_centrality
Information_centrality
Degree_centrality
Load_centrality
Page_rank
Betweenness_centrality
Centrality

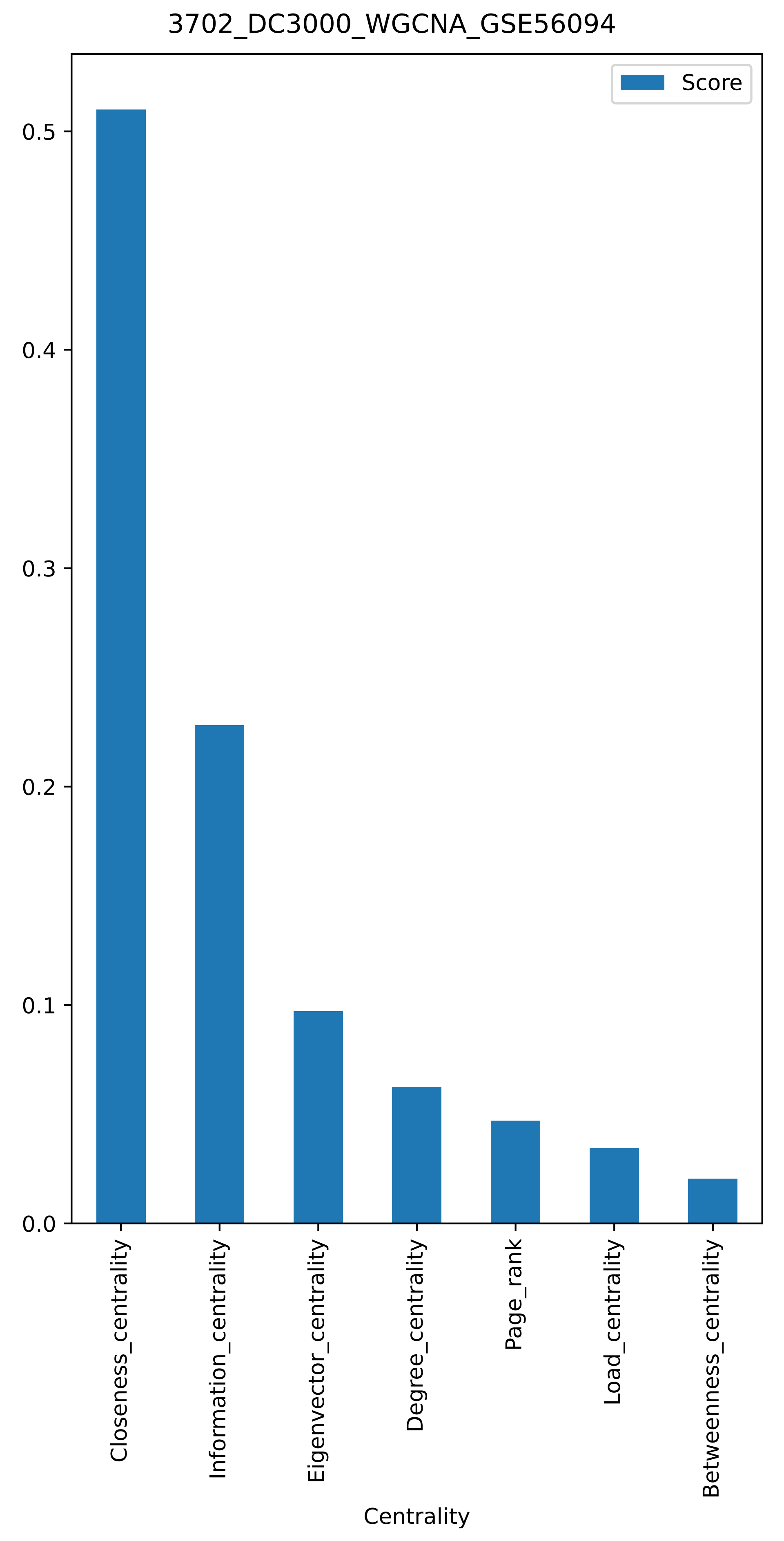
3702_DC3000_WGCNA_GSE56094
Score
0.5
0.4
0.3
0.2
0.1
0.0
Closeness_centrality
Information_centrality
Eigenvector_centrality
Degree_centrality
Page_rank
Load_centrality
Betweenness_centrality
Centrality

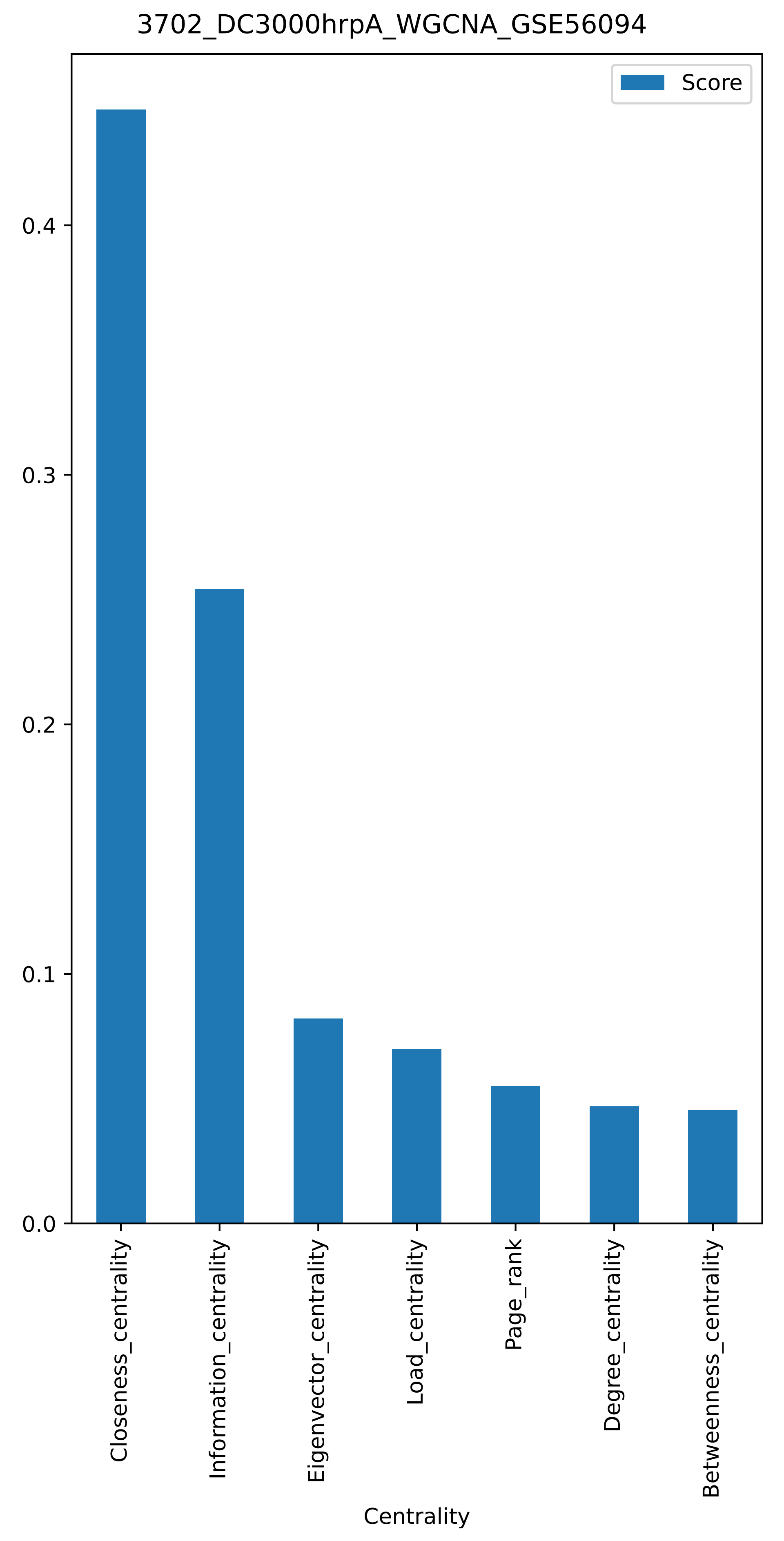
3702_DC3000hrpA_WGCNA_GSE56094
Score
0.0
0.1
0.2
0.3
0.4
Closeness_centrality
Information_centrality
Eigenvector_centrality
Load_centrality
Page_rank
Degree_centrality
Betweenness_centrality
Centrality

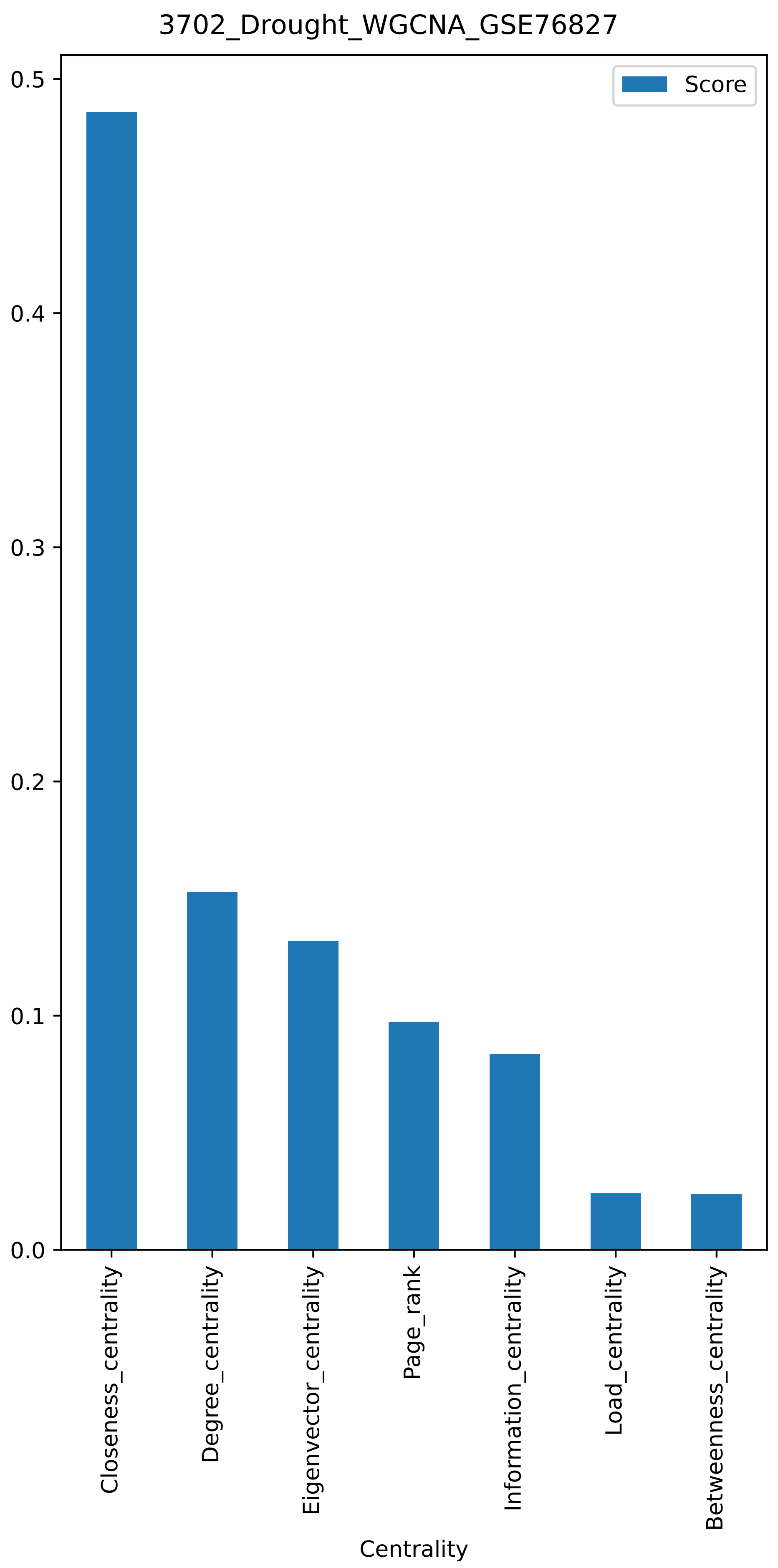

3702_Drought_WGCNA_GSE76827
Score
0.5
0.4
0.3
0.2
0.1
0.0
Closeness_centrality
Degree_centrality
Eigenvector_centrality
Page_rank
Information_centrality
Load_centrality
Betweenness_centrality
Centrality

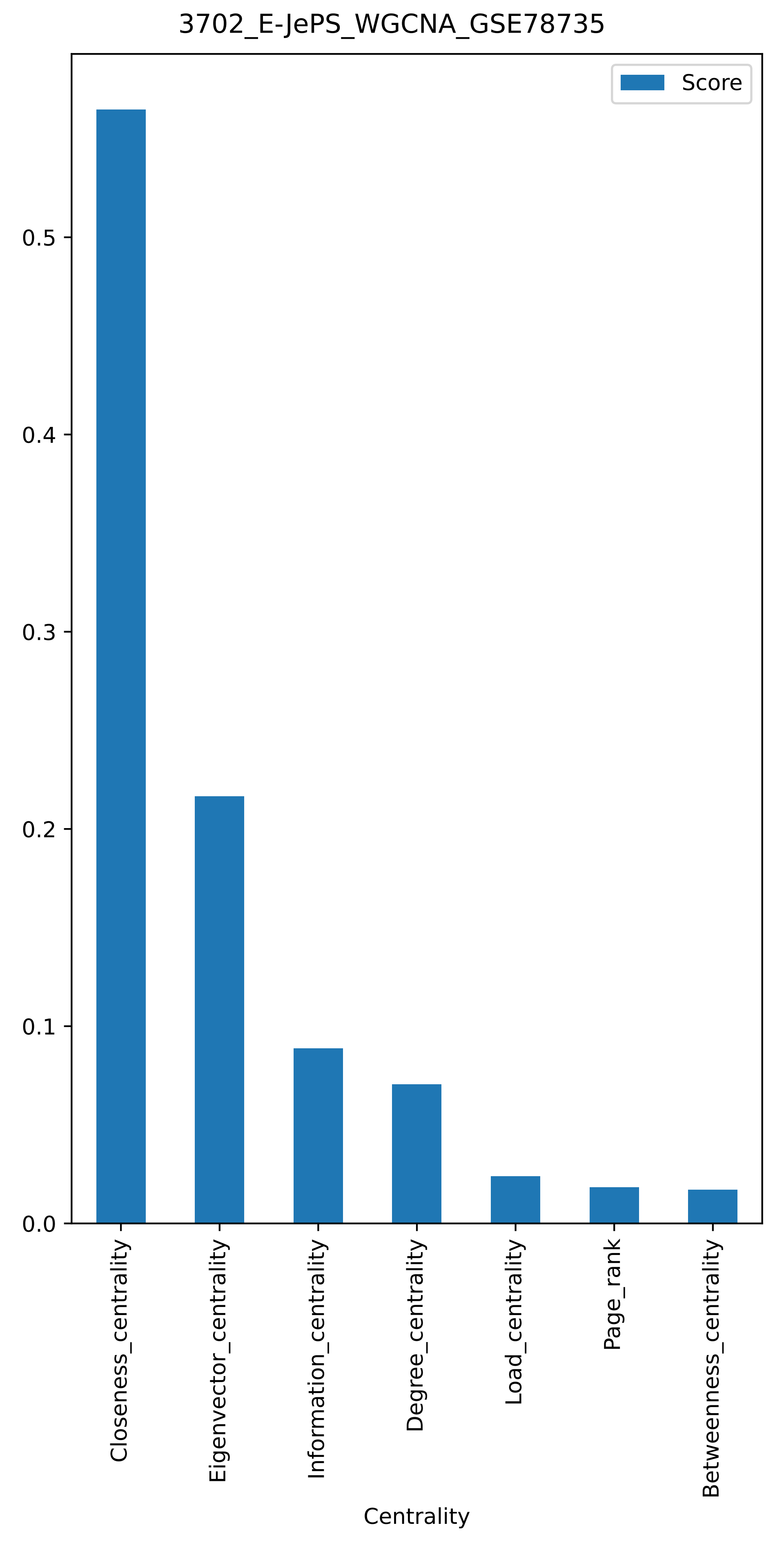
3702_E-JePS_WGCNA_GSE78735
Score
0.0
0.1
0.2
0.3
0.4
0.5
Closeness_centrality
Eigenvector_centrality
Information_centrality
Degree_centrality
Load_centrality
Page_rank
Betweenness_centrality
Centrality

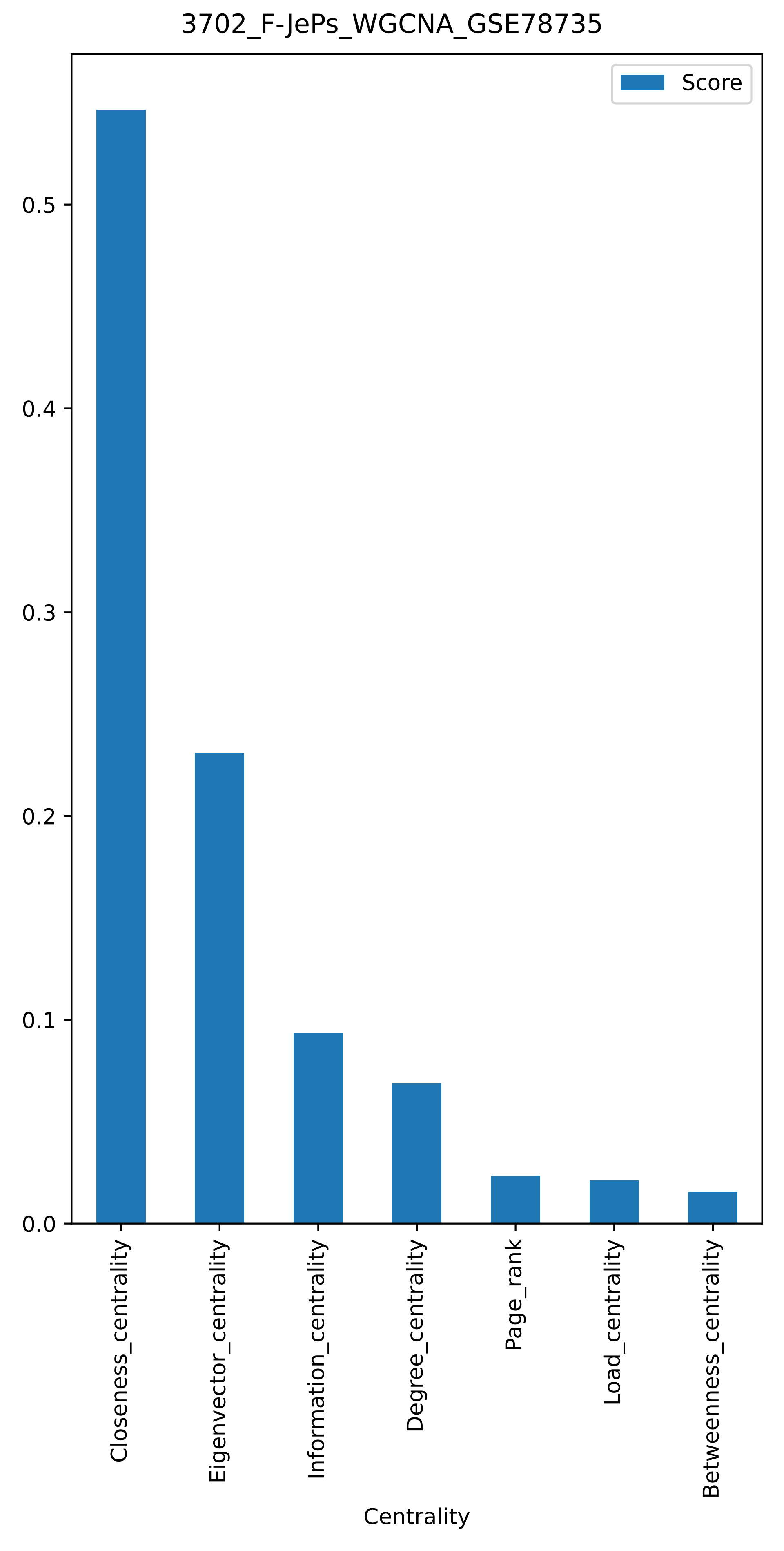
3702_F-JePs_WGCNA_GSE78735
Score
0.5
0.4
0.3
0.2
0.1
0.0
Closeness_centrality
Eigenvector_centrality
Information_centrality
Degree_centrality
Page_rank
Load_centrality
Betweenness_centrality
Centrality

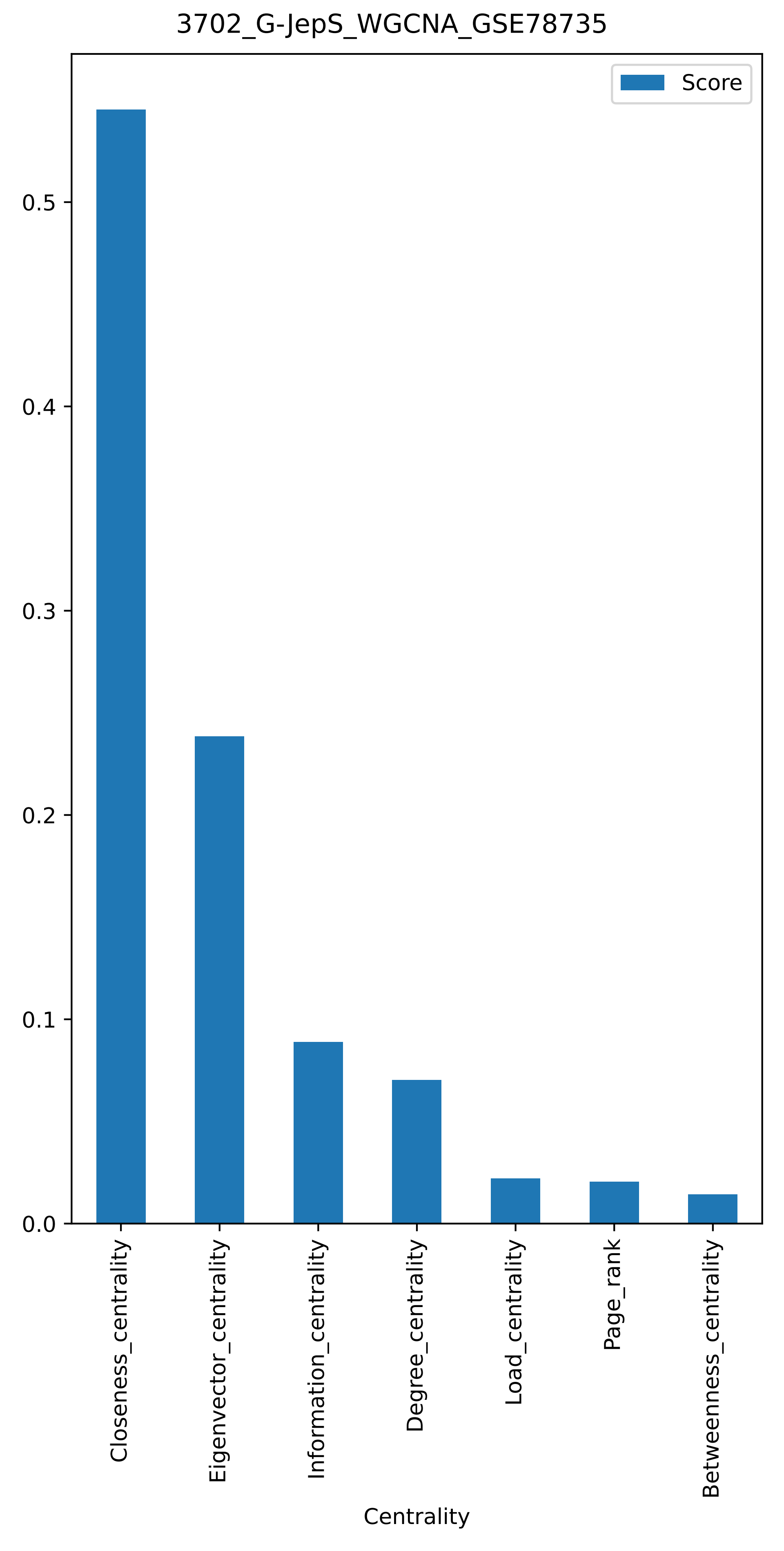
3702_G-JepS_WGCNA_GSE78735
Score
0.5
0.4
0.3
0.2
0.1
0.0
Closeness_centrality
Eigenvector_centrality
Information_centrality
Degree_centrality
Load_centrality
Page_rank
Betweenness_centrality
Centrality

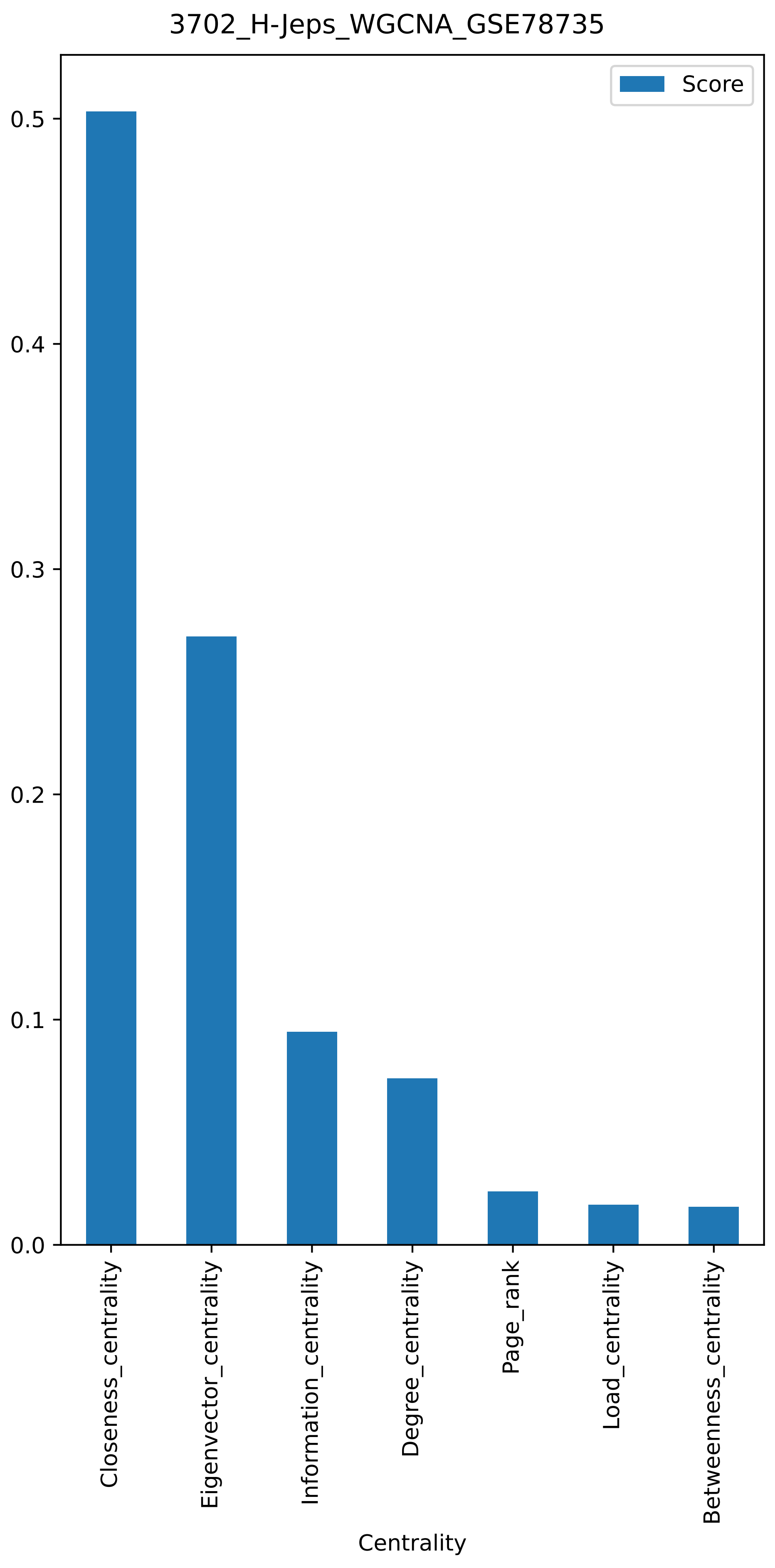

3702_H-Jeps_WGCNA_GSE78735
Score
0.5
0.4
0.3
0.2
0.1
0.0
Closeness_centrality
Eigenvector_centrality
Information_centrality
Degree_centrality
Page_rank
Load_centrality
Betweenness_centrality
Centrality

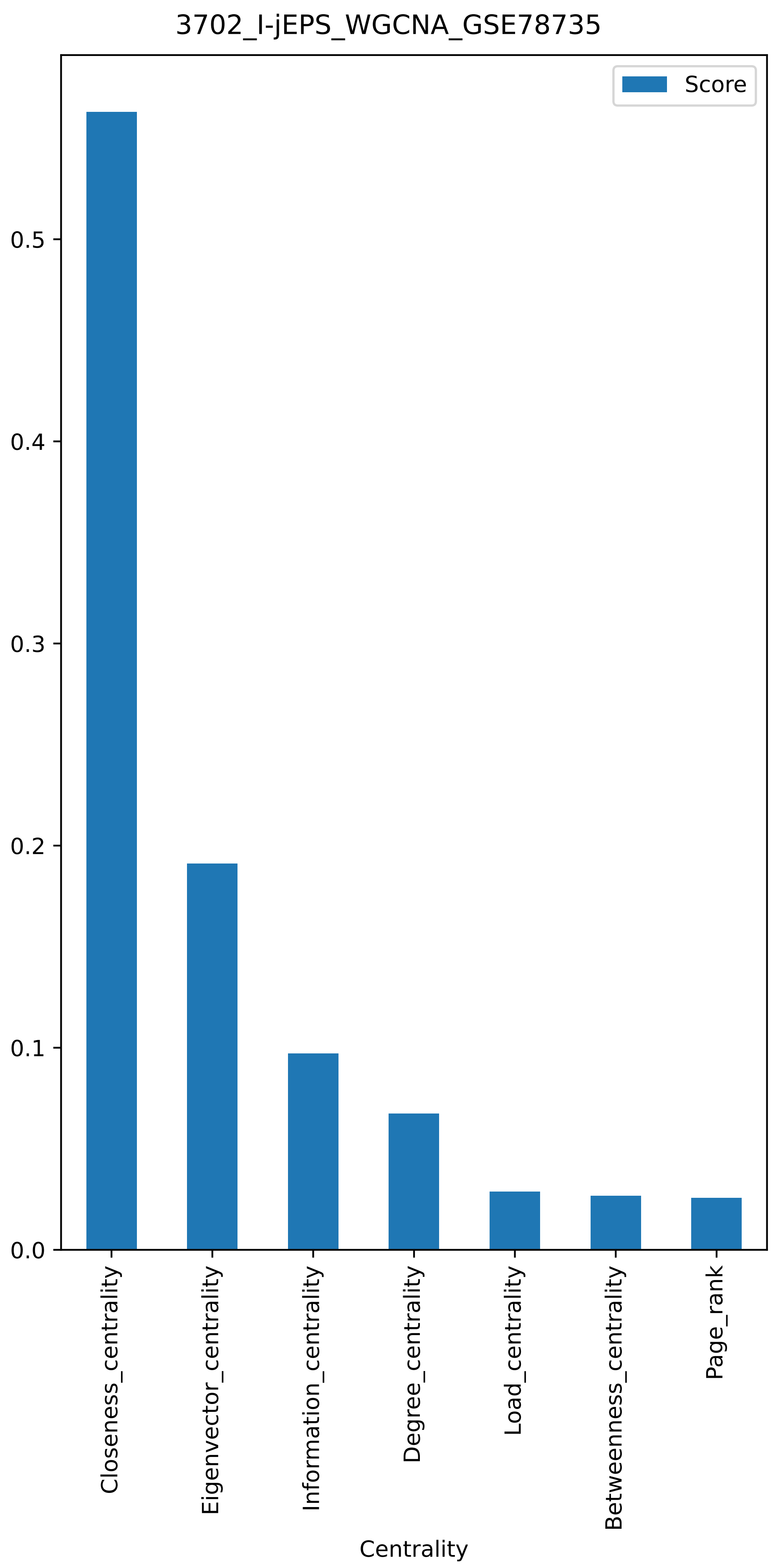

3702_I-jEPS_WGCNA_GSE78735
Score
0.0
0.1
0.2
0.3
0.4
0.5
Closeness_centrality
Eigenvector_centrality
Information_centrality
Degree_centrality
Load_centrality
Betweenness_centrality
Page_rank
Centrality

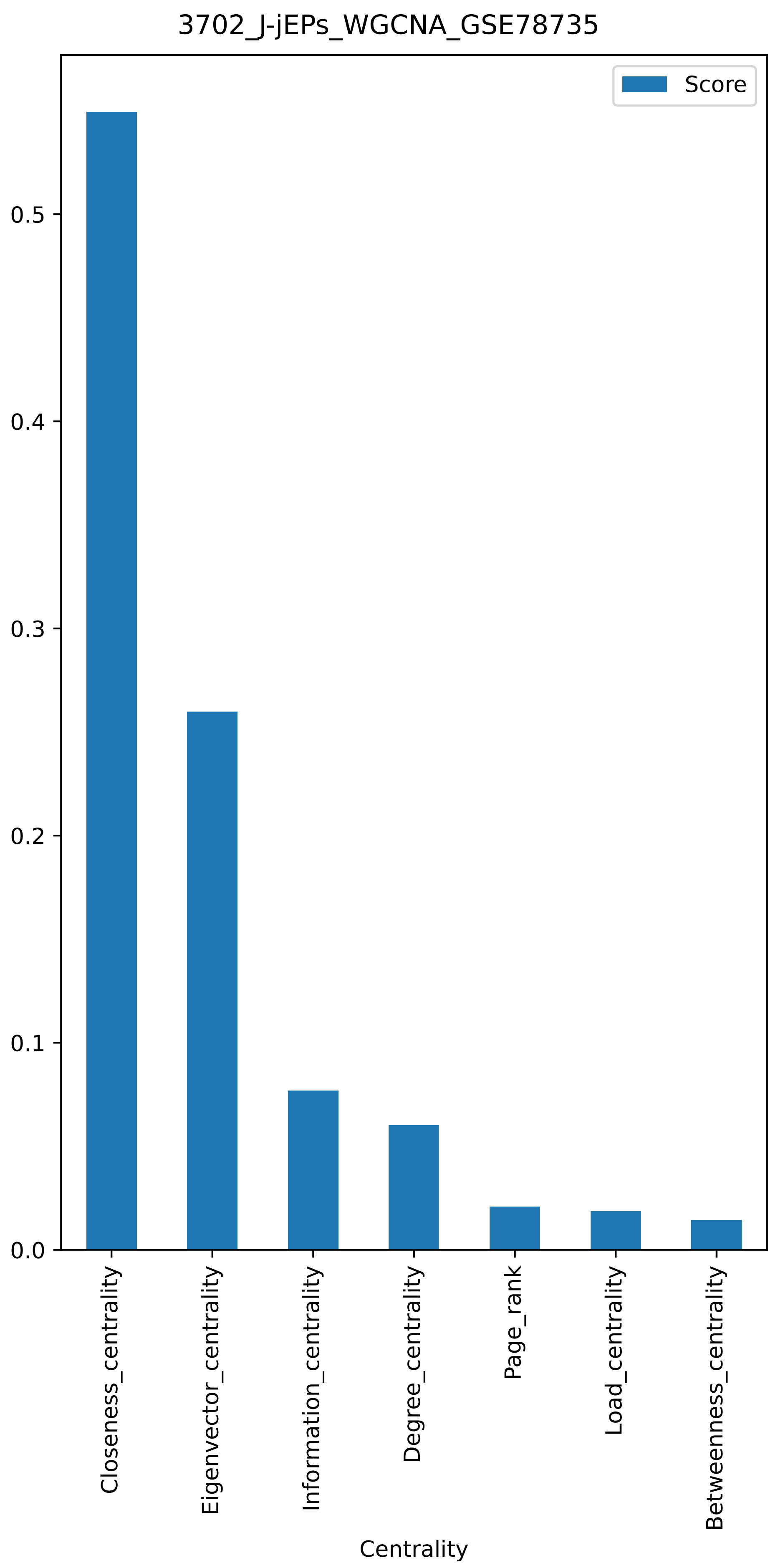
3702_J-jEPs_WGCNA_GSE78735
Score
0.5
0.4
0.3
0.2
0.1
0.0
Closeness_centrality
Eigenvector_centrality
Information_centrality
Degree_centrality
Page_rank
Load_centrality
Betweenness_centrality
Centrality

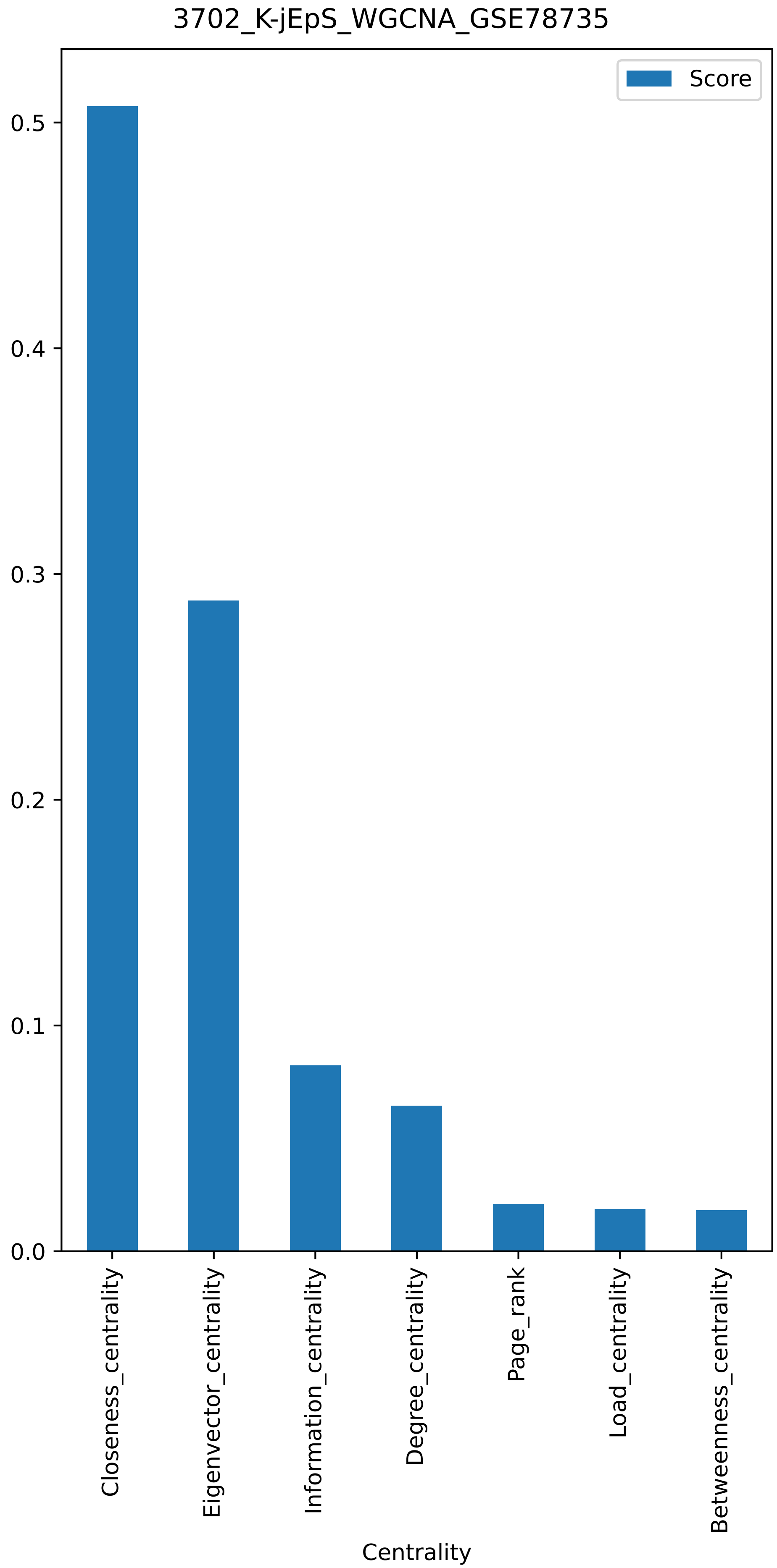
3702_K-jEpS_WGCNA_GSE78735
Score
0.5
0.4
0.3
0.2
0.1
0.0
Closeness_centrality
Eigenvector_centrality
Information_centrality
Degree_centrality
Page_rank
Load_centrality
Betweenness_centrality
Centrality

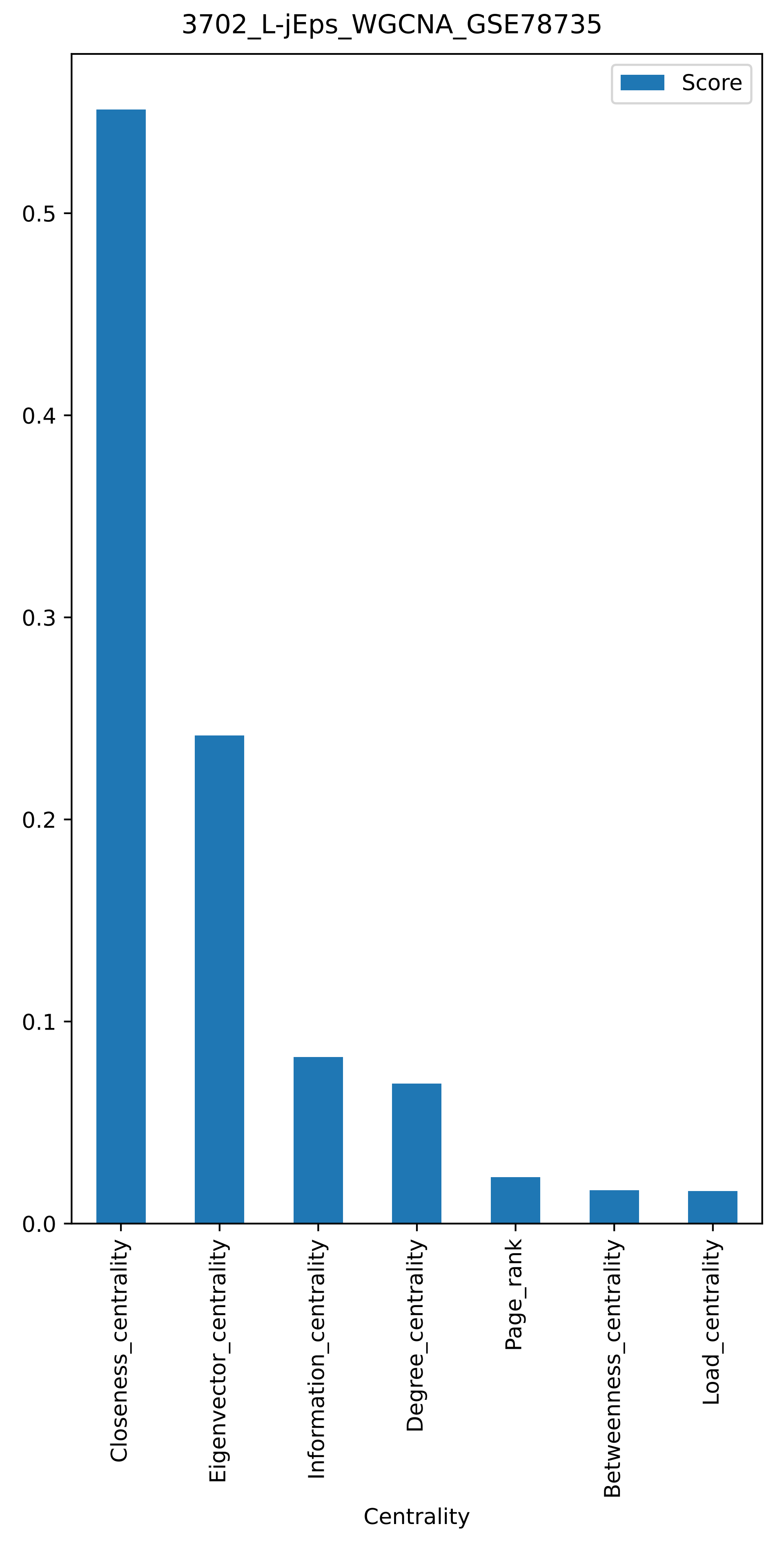

3702_L-jEps_WGCNA_GSE78735
Score
0.0
0.1
0.2
0.3
0.4
0.5
Closeness_centrality
Eigenvector_centrality
Information_centrality
Degree_centrality
Page_rank
Betweenness_centrality
Load_centrality
Centrality

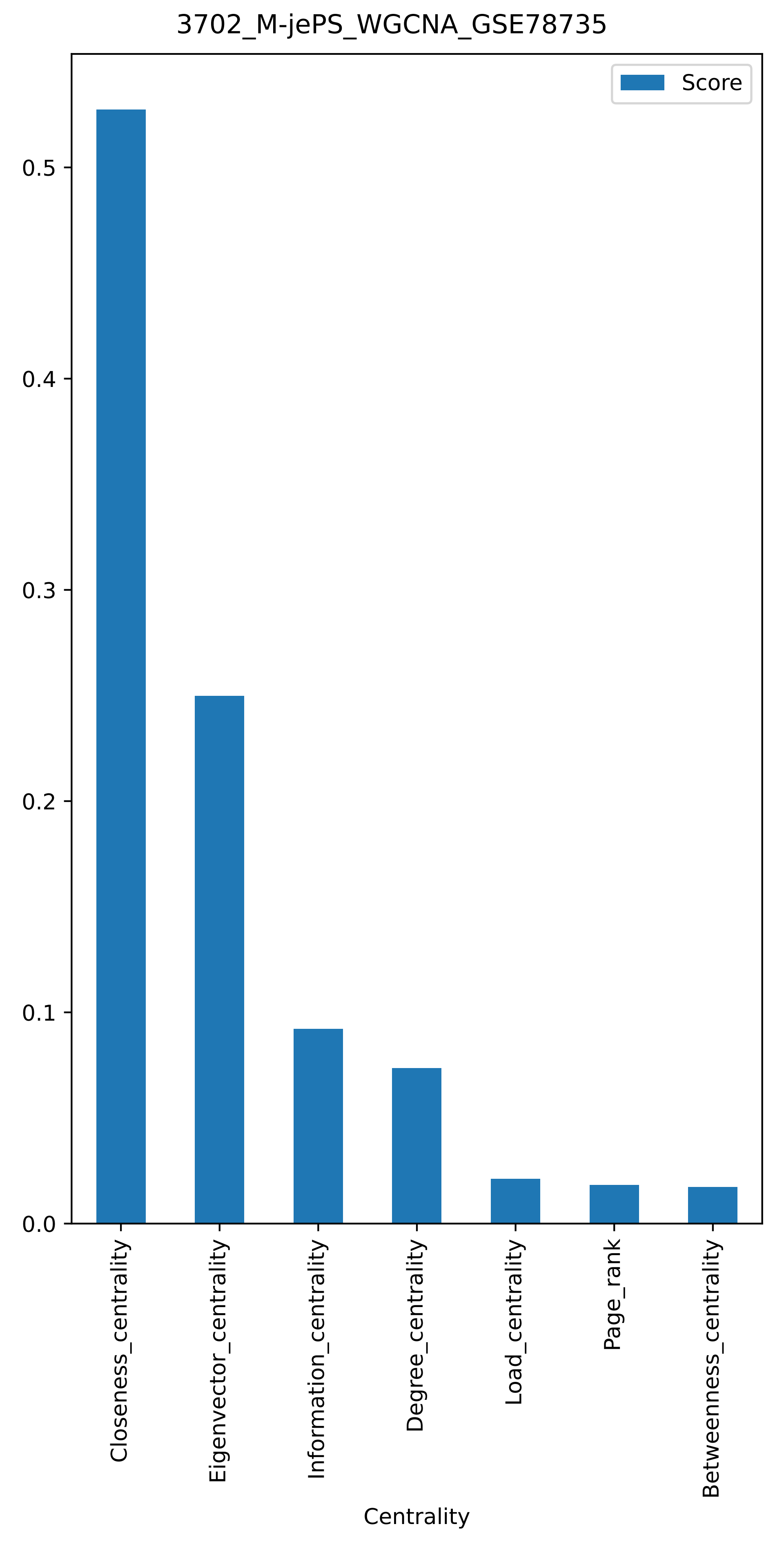

3702_M-jePS_WGCNA_GSE78735
Score
0.0
0.1
0.2
0.3
0.4
0.5
Closeness_centrality
Eigenvector_centrality
Information_centrality
Degree_centrality
Load_centrality
Page_rank
Betweenness_centrality
Centrality

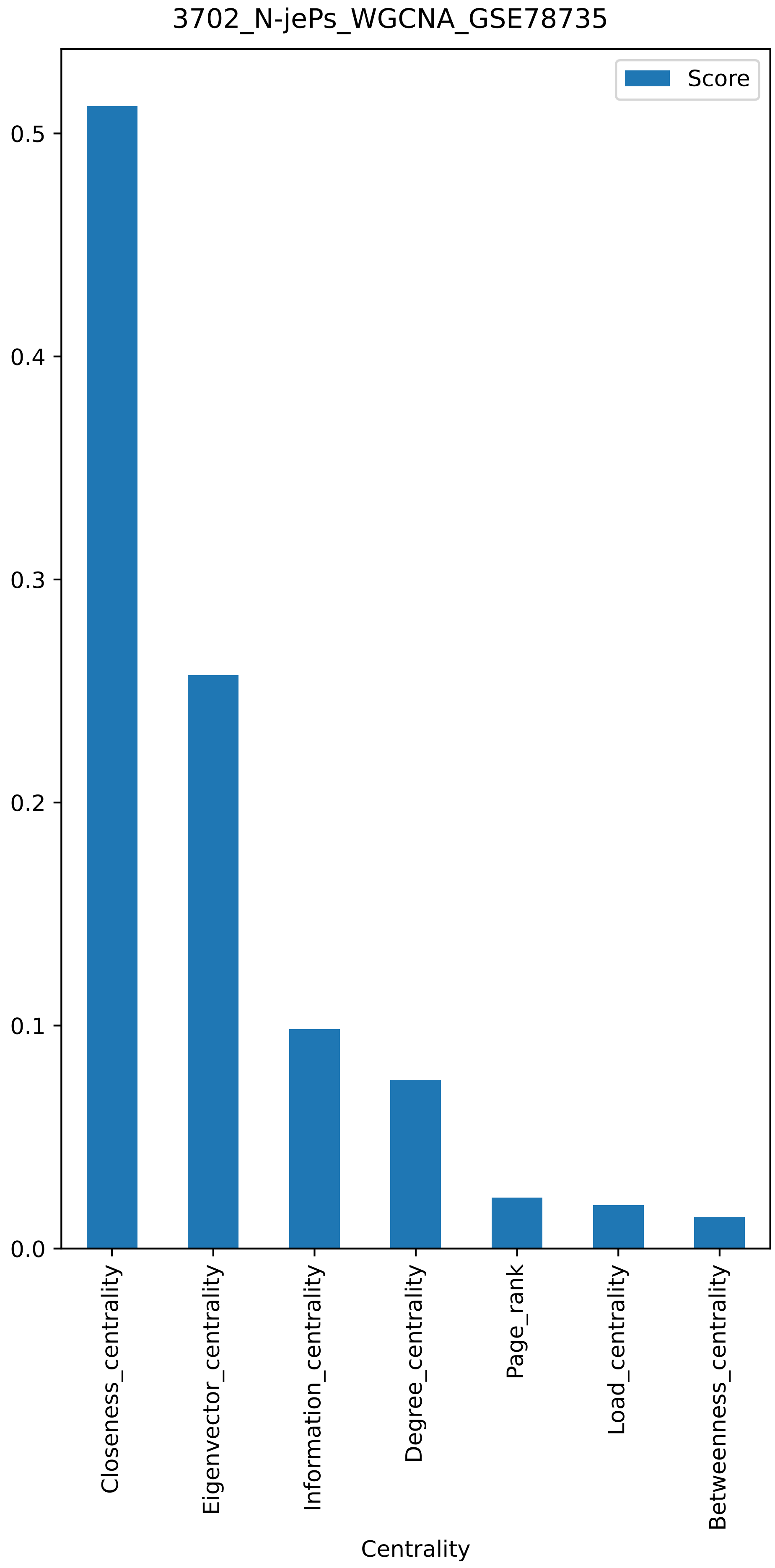

3702_N-jePs_WGCNA_GSE78735
Score
0.0
0.1
0.2
0.3
0.4
0.5
Closeness_centrality
Eigenvector_centrality
Information_centrality
Degree_centrality
Page_rank
Load_centrality
Betweenness_centrality
Centrality

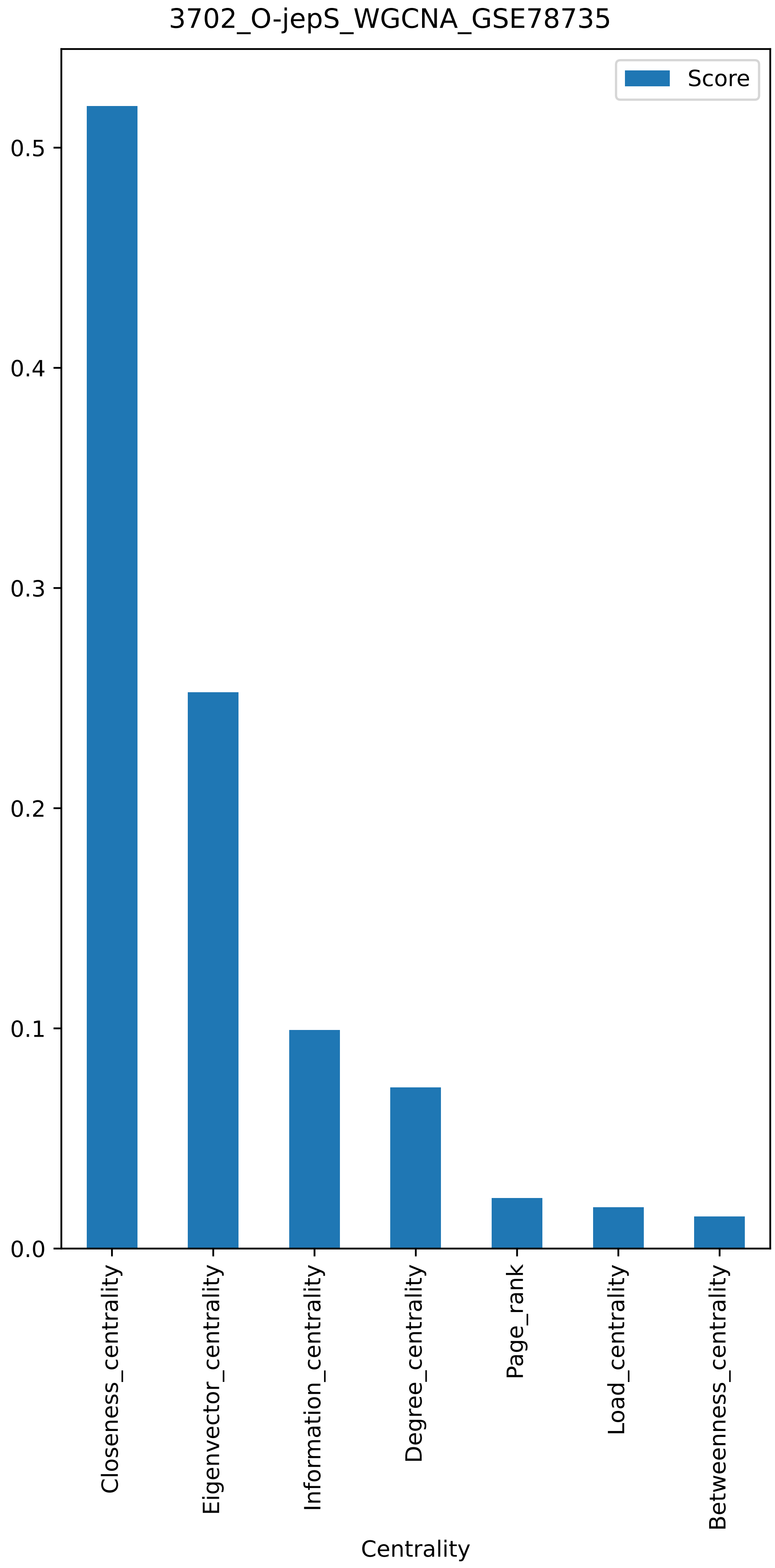
3702_O-jepS_WGCNA_GSE78735
Score
0.0
0.1
0.2
0.3
0.4
0.5
Closeness_centrality
Eigenvector_centrality
Information_centrality
Degree_centrality
Page_rank
Load_centrality
Betweenness_centrality
Centrality

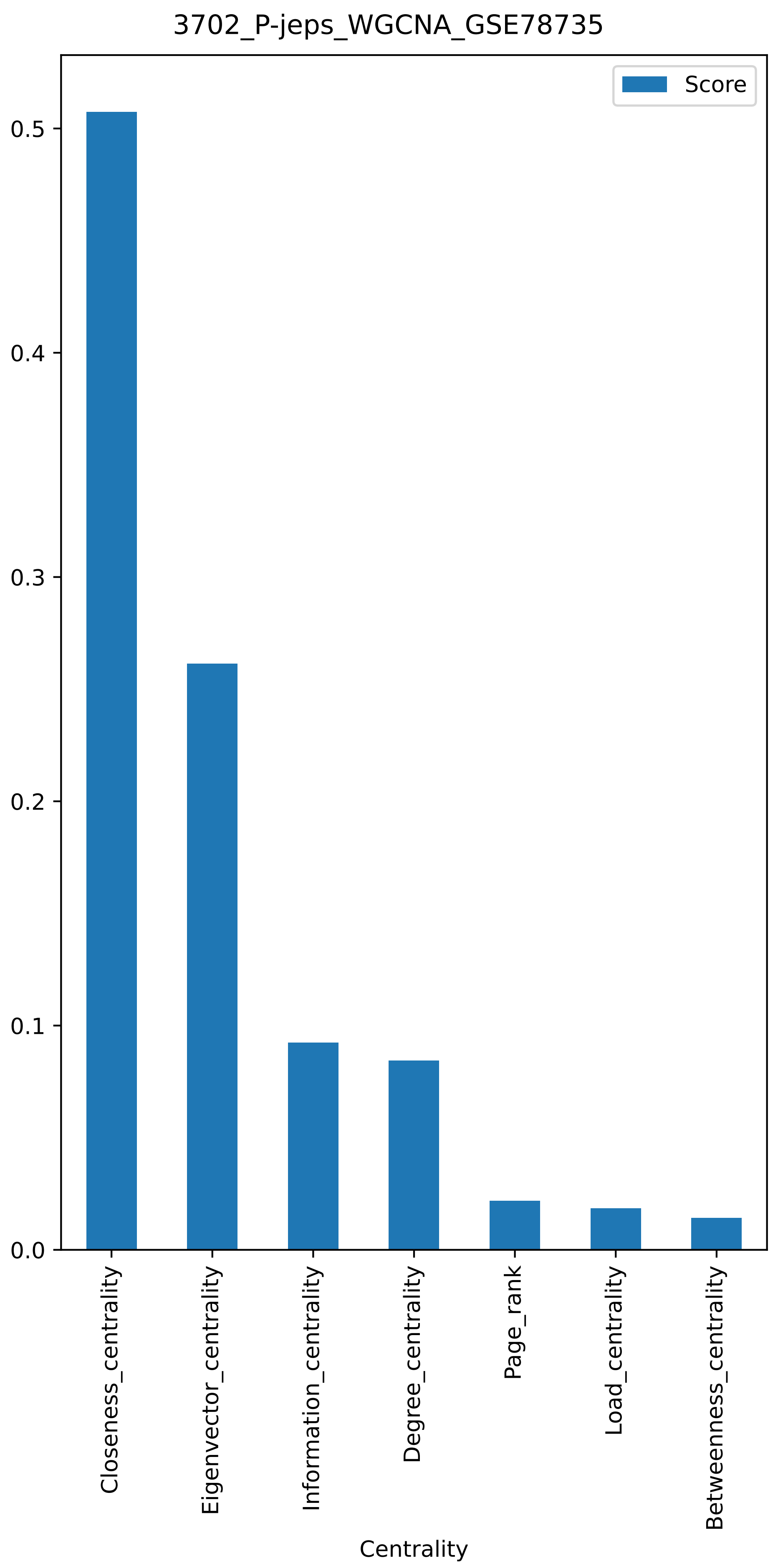
3702_P-jeps_WGCNA_GSE78735
Score
0.5
0.4
0.3
0.2
0.1
0.0
Closeness_centrality
Eigenvector_centrality
Information_centrality
Degree_centrality
Page_rank
Load_centrality
Betweenness_centrality
Centrality

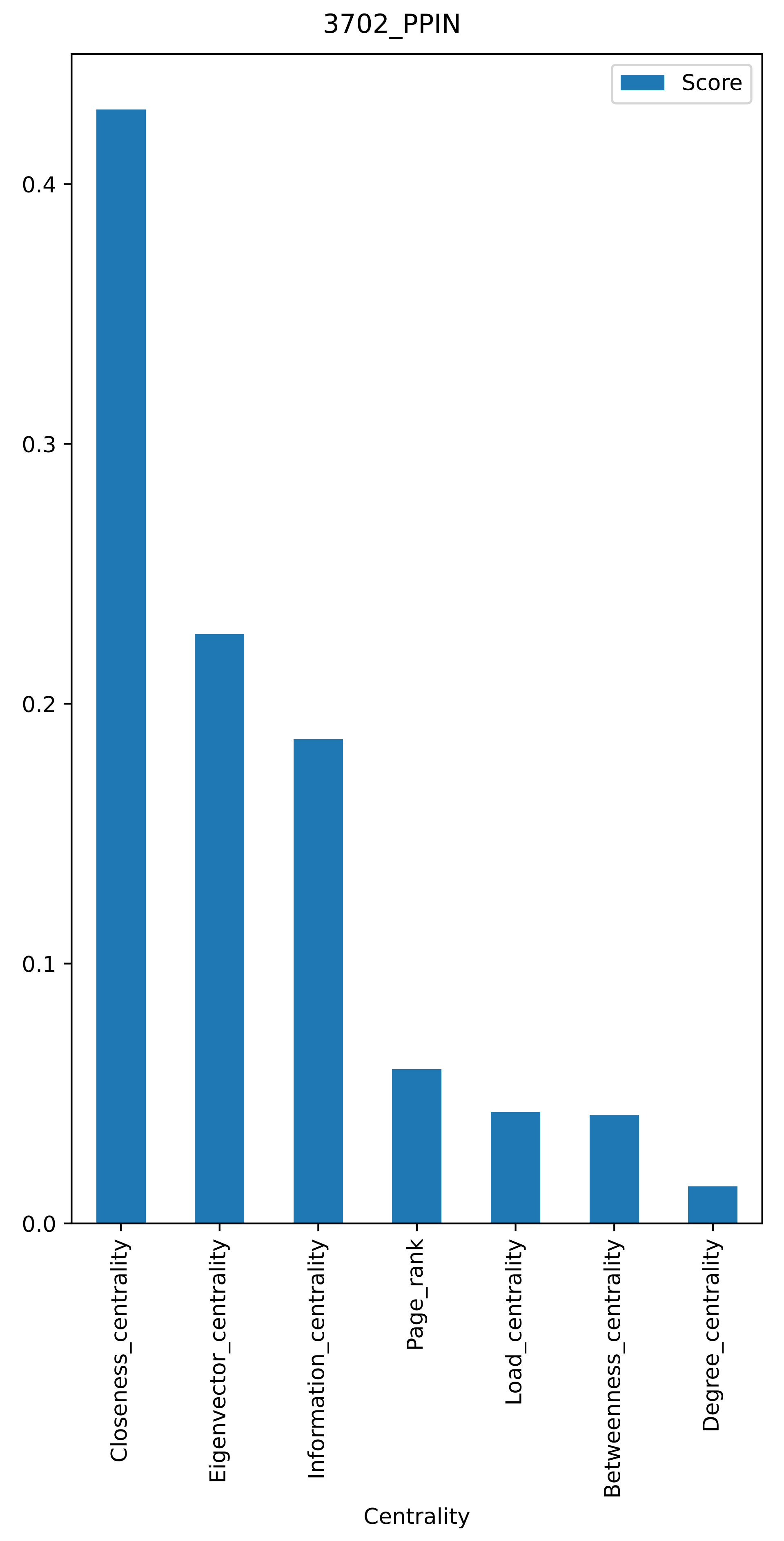
3702_PPIN
Score
0.0
0.1
0.2
0.3
0.4
Closeness_centrality
Eigenvector_centrality
Information_centrality
Page_rank
Load_centrality
Betweenness_centrality
Degree_centrality
Centrality

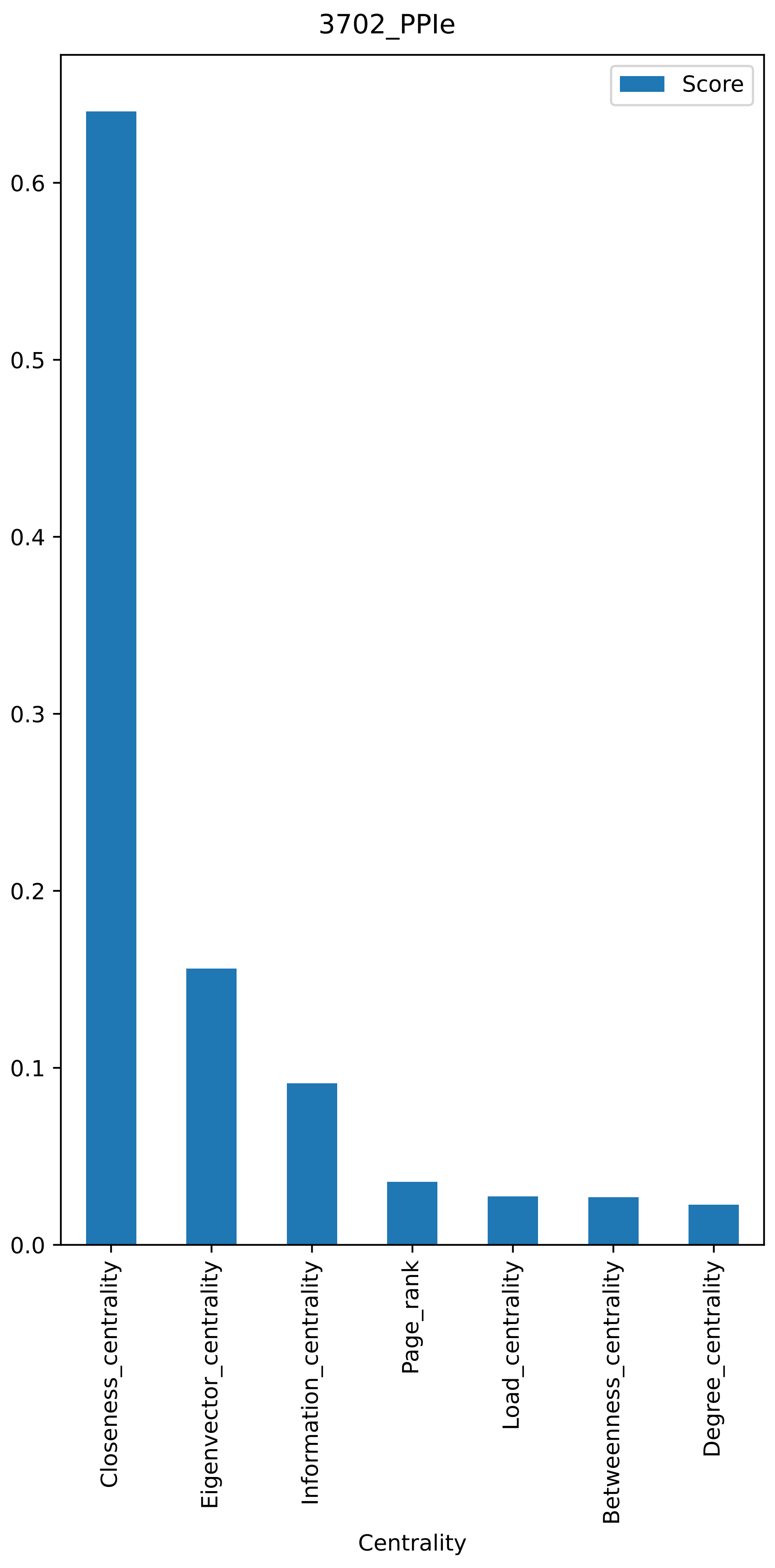
3702_PPIe
Score
0.0
0.1
0.2
0.3
0.4
0.5
0.6
Closeness_centrality
Eigenvector_centrality
Information_centrality
Page_rank
Load_centrality
Betweenness_centrality
Degree_centrality
Centrality

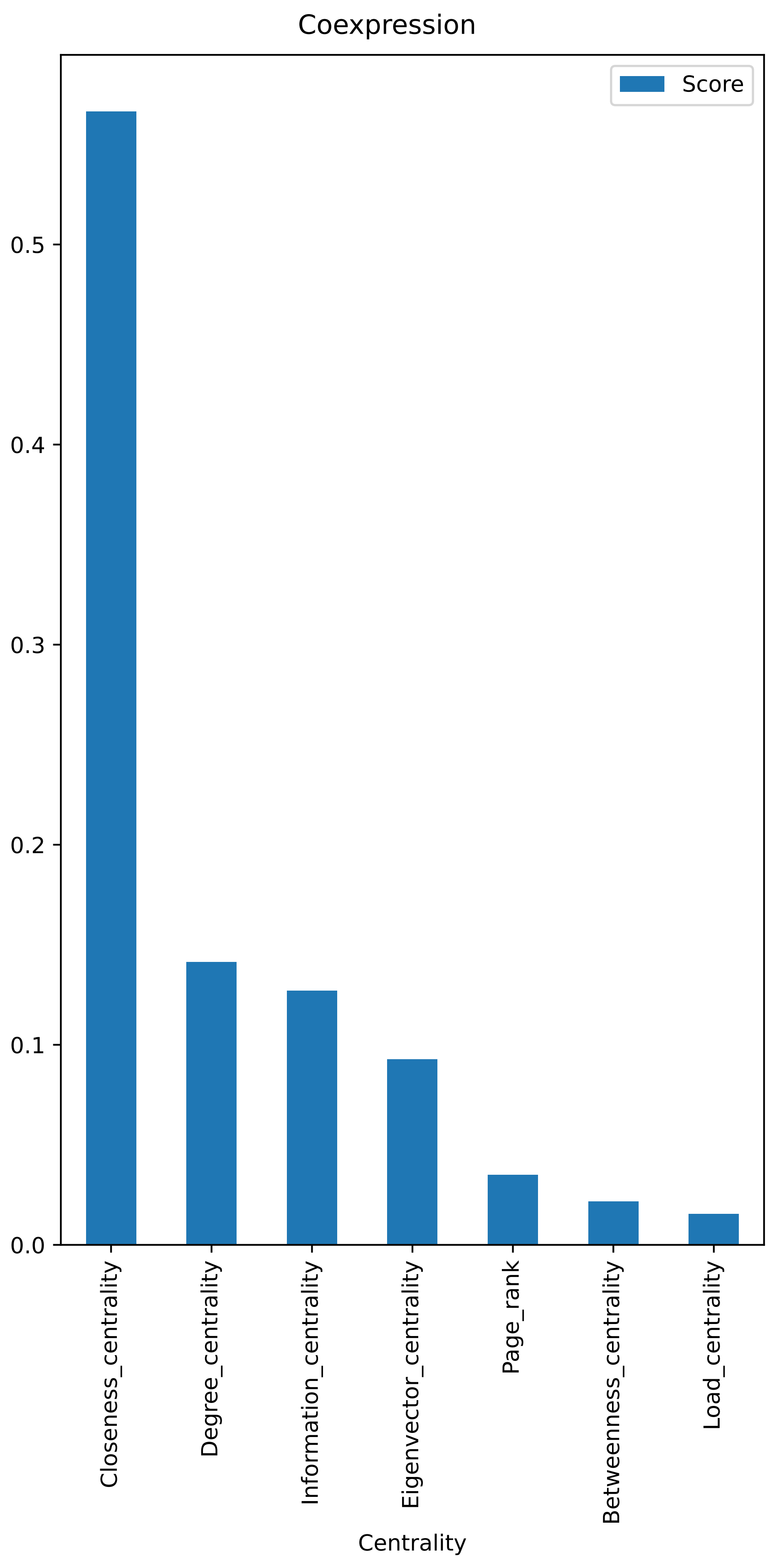
Coexpression
Score
0.0
0.1
0.2
0.3
0.4
0.5
Closeness_centrality
Degree_centrality
Information_centrality
Eigenvector_centrality
Page_rank
Betweenness_centrality
Load_centrality
Centrality

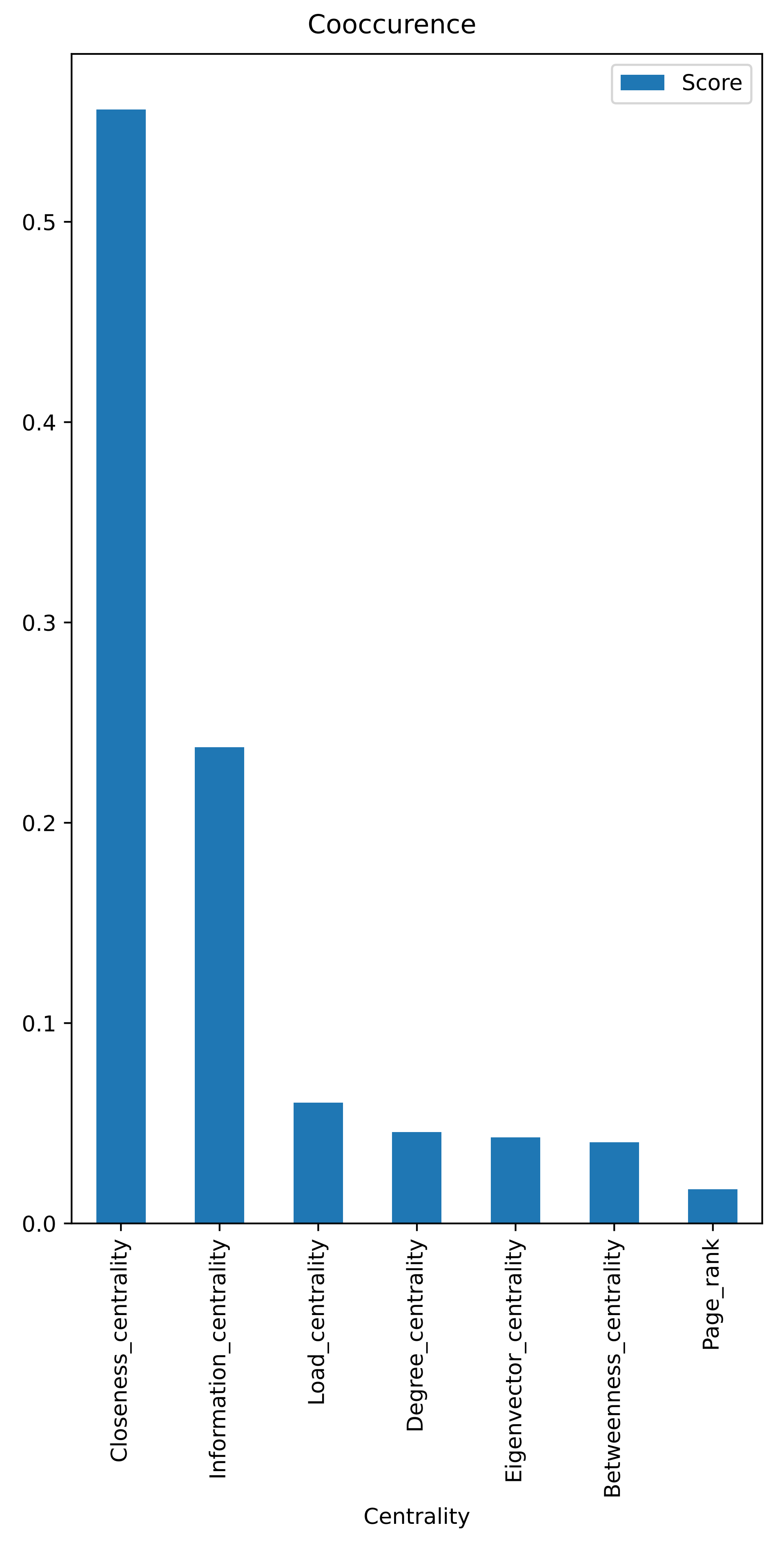

Cooccurence
Score
0.0
0.1
0.2
0.3
0.4
0.5
Closeness_centrality
Information_centrality
Load_centrality
Degree_centrality
Eigenvector_centrality
Betweenness_centrality
Page_rank
Centrality

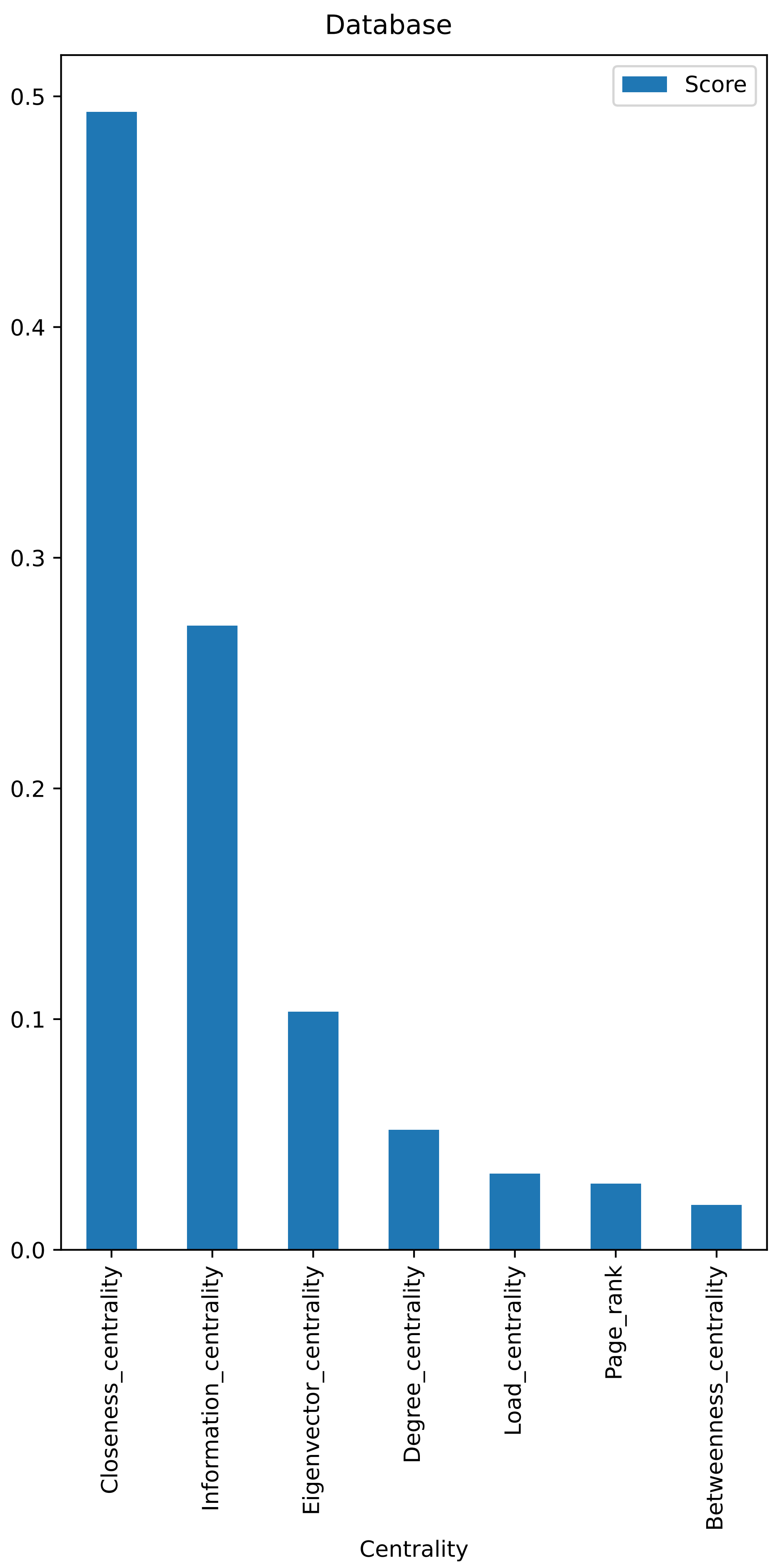

Database
Score
0.5
0.4
0.3
0.2
0.1
0.0
Closeness_centrality
Information_centrality
Eigenvector_centrality
Degree_centrality
Load_centrality
Page_rank
Betweenness_centrality
Centrality

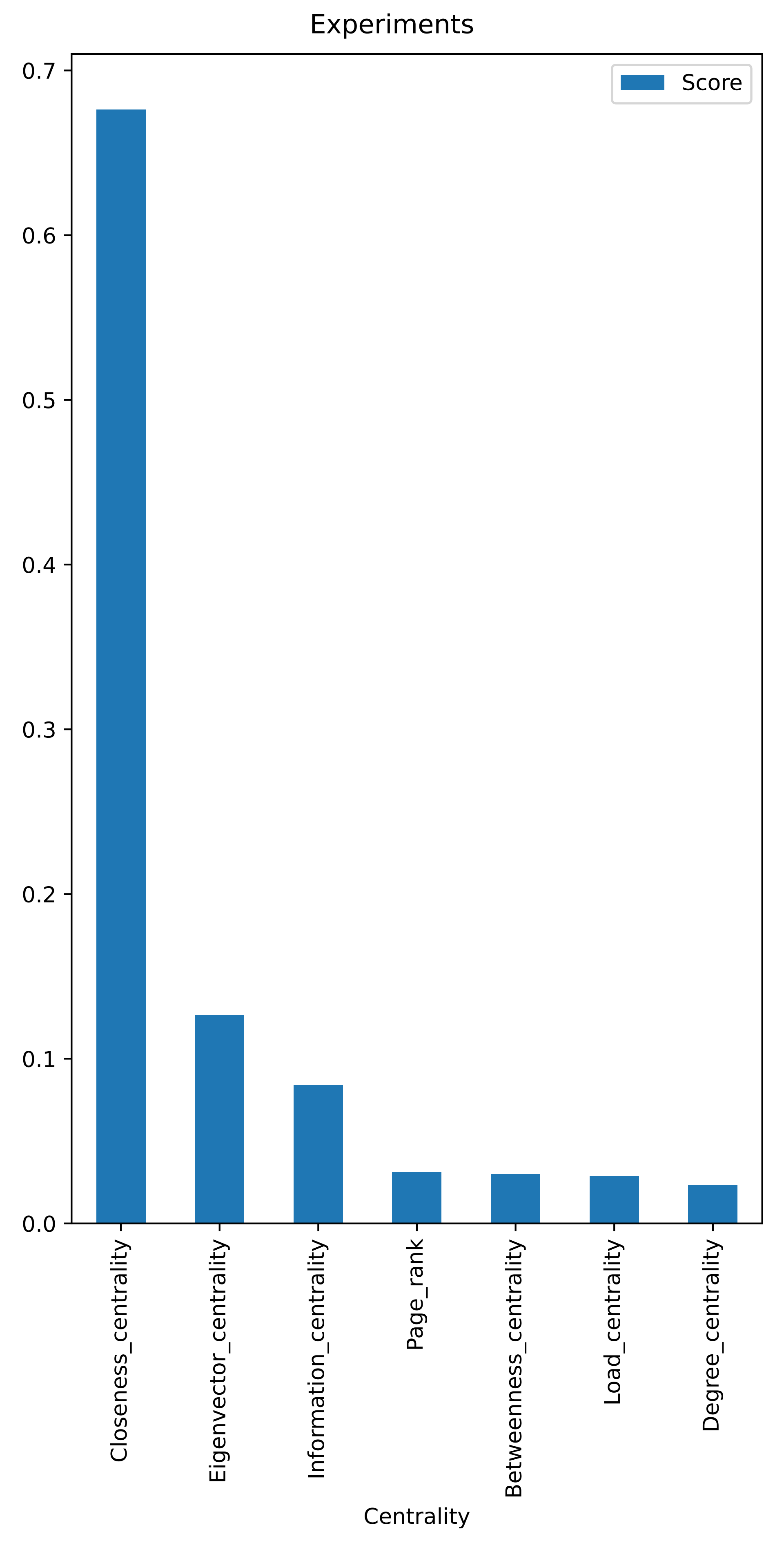

Experiments
Score
0.7
0.6
0.5
0.4
0.3
0.2
0.1
0.0
Closeness_centrality
Eigenvector_centrality
Information_centrality
Page_rank
Betweenness_centrality
Load_centrality
Degree_centrality
Centrality

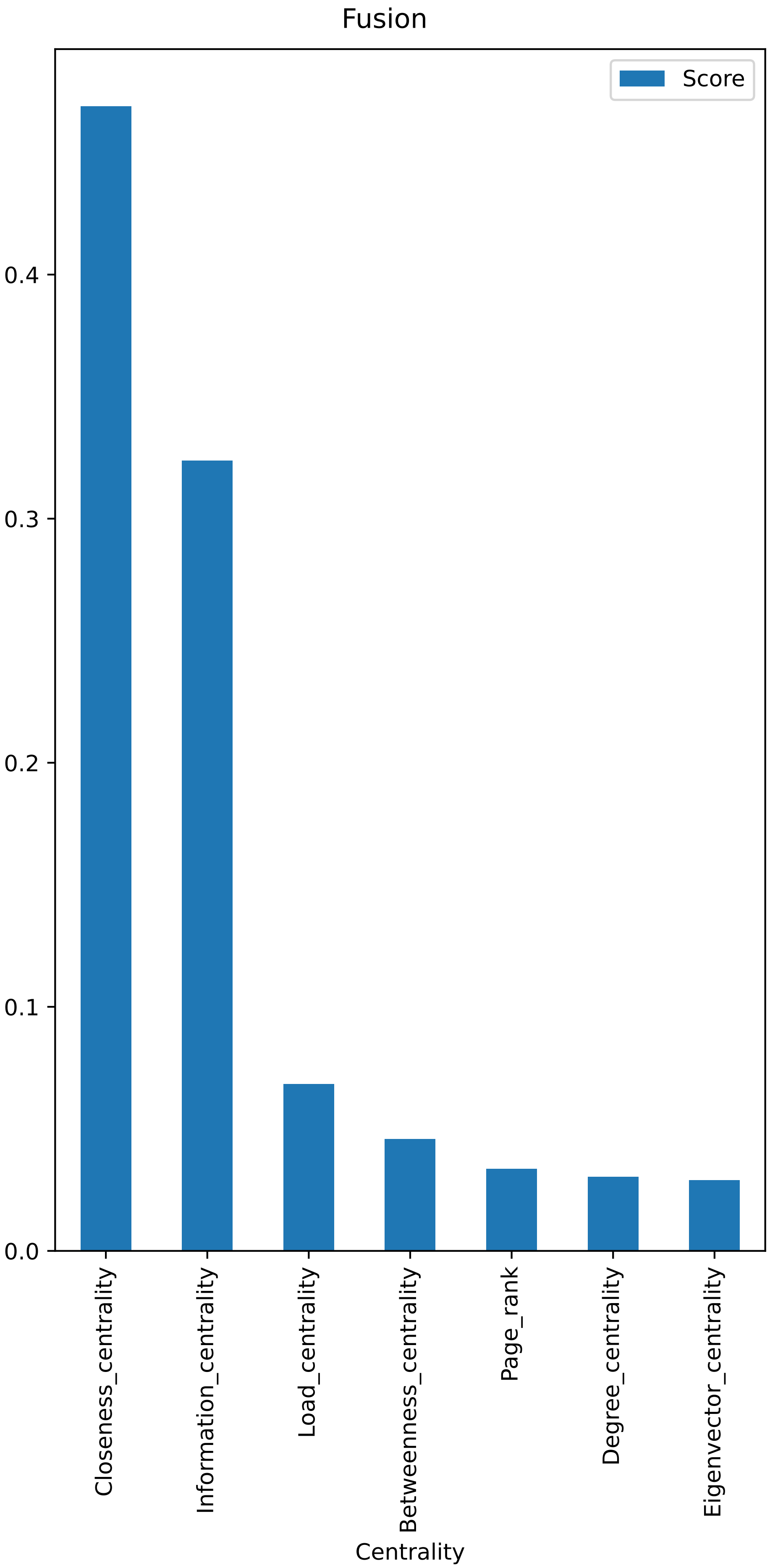

Fusion
Score
0.0
0.1
0.2
0.3
0.4
Closeness_centrality
Information_centrality
Load_centrality
Betweenness_centrality
Page_rank
Degree_centrality
Eigenvector_centrality
Centrality

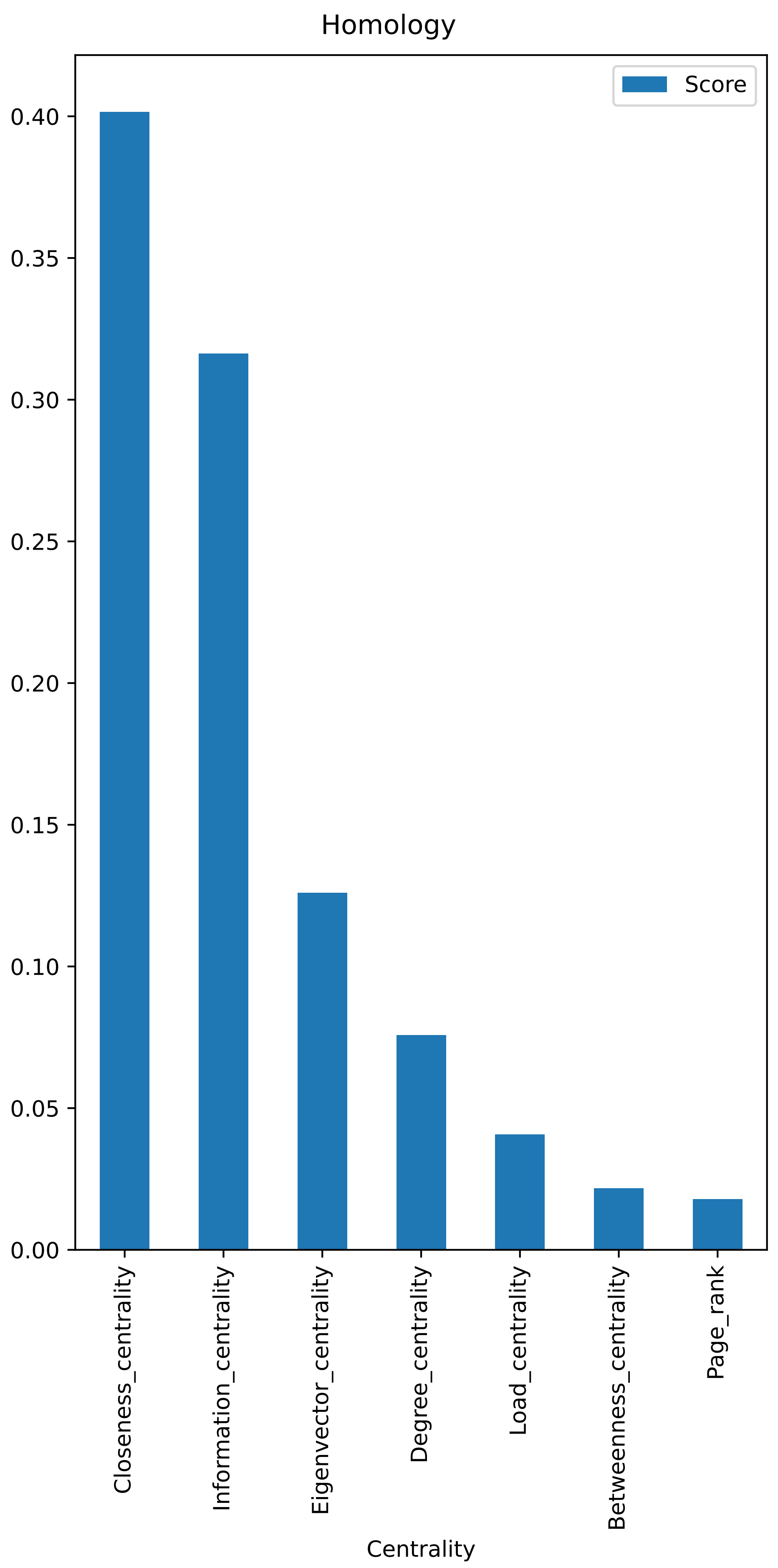

Homology
Score
0.00
0.05
0.10
0.15
0.20
0.25
0.30
0.35
0.40
Closeness_centrality
Information_centrality
Eigenvector_centrality
Degree_centrality
Load_centrality
Betweenness_centrality
Page_rank
Centrality

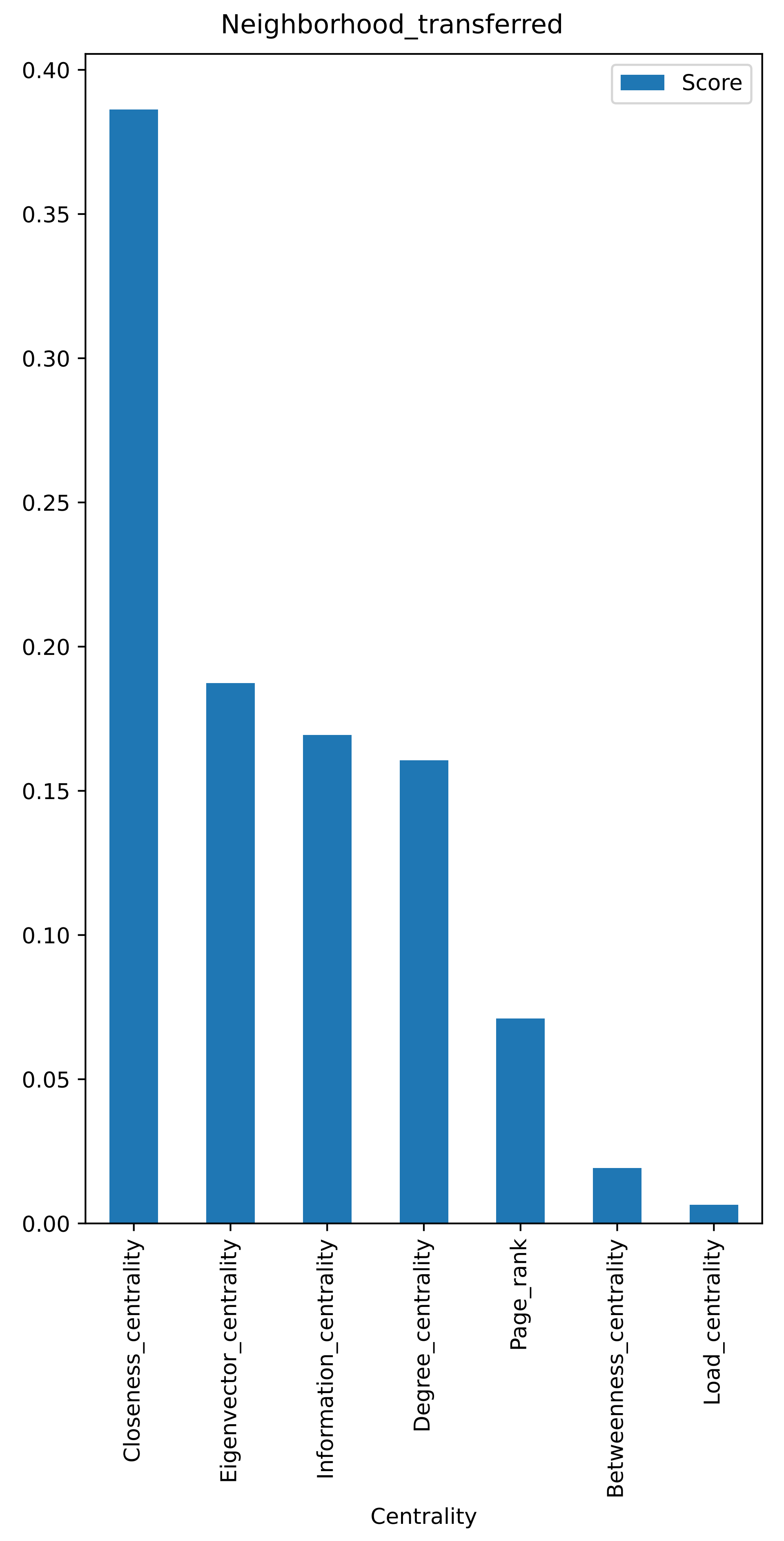
Neighborhood_transferred
Score
0.40
0.35
0.30
0.25
0.20
0.15
0.10
0.05
0.00
Closeness_centrality
Eigenvector_centrality
Information_centrality
Degree_centrality
Page_rank
Betweenness_centrality
Load_centrality
Centrality

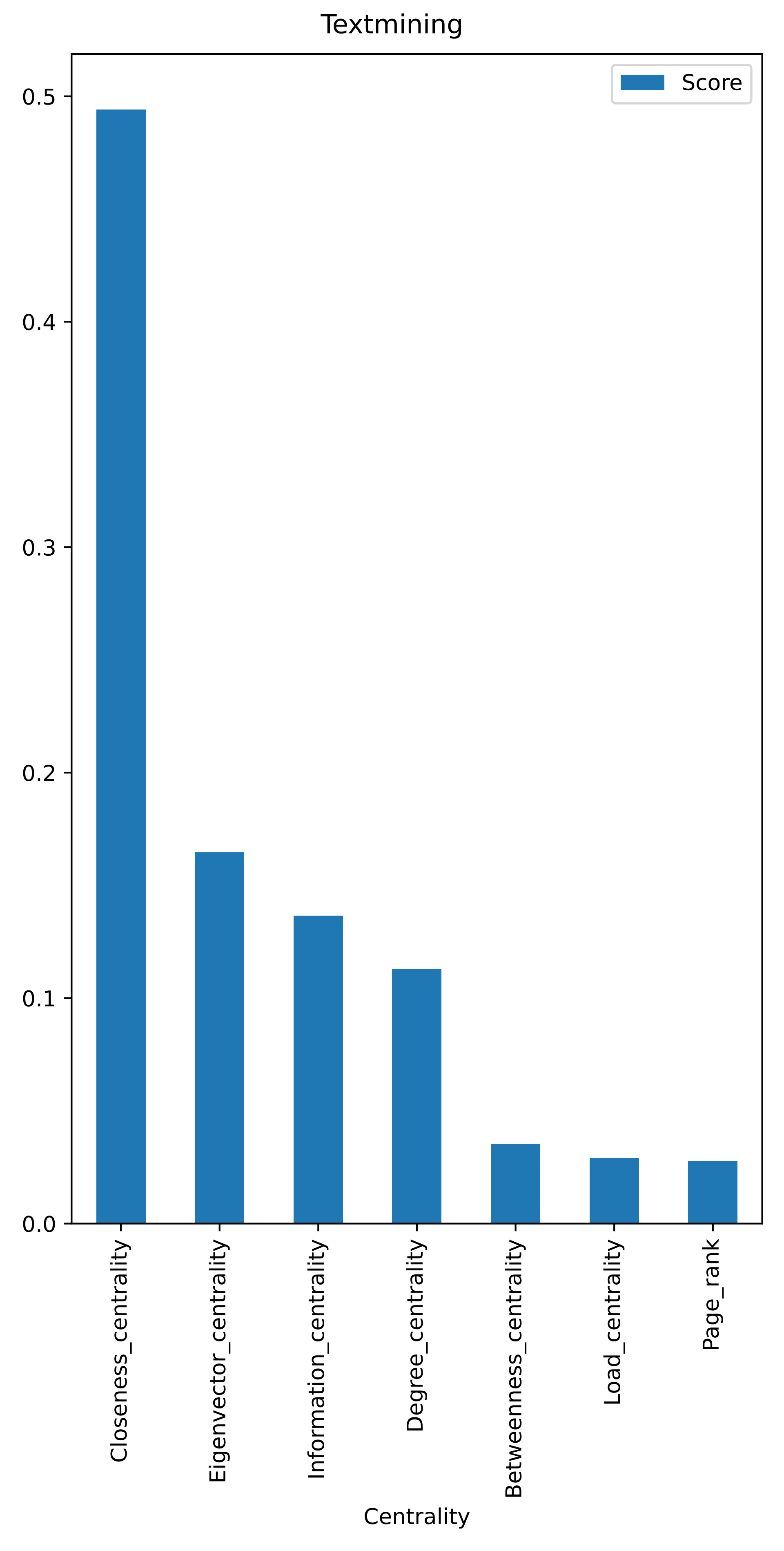
Textmining
Score
0.5
0.4
0.3
0.2
0.1
0.0
Closeness_centrality
Eigenvector_centrality
Information_centrality
Degree_centrality
Betweenness_centrality
Load_centrality
Page_rank
Centrality
